# Soil-Matrix-Dependent Root Reinforcement by *Chrysopogon zizanioides*: Direct Shear and Statistical Evidence from Andean Soils

**DOI:** 10.3390/plants15152328

**Published:** 2026-07-29

**Authors:** Jose Luis Chavez-Torres, Kunyong Zhang, Camila Nickole Fernandez-Morocho

**Affiliations:** 1 Department of Civil Engineering, Universidad Técnica Particular de Loja, Loja 1101608, Ecuador; 2 College of Civil and Transportation Engineering, Hohai University, Nanjing 210024, China; 3 Research Institute of Geotechnical Engineering, Hohai University, Nanjing 210024, China; 4 Key Laboratory of Ministry of Education for Geomechanics and Embankment Engineering, Hohai University, Nanjing 210024, China

**Keywords:** *Chrysopogon zizanioides*, Vetiver grass, soil–root interaction, root reinforcement, biomechanical reinforcement, soil matrix, apparent cohesion, internal friction angle, direct shear test, Andean soils

## Abstract

Plant roots can modify soil mechanical behaviour through anchorage, interlocking, and stress-transfer mechanisms; however, the magnitude of this effect is not uniform and depends on the soil matrix in which the root system develops. This study evaluates the biomechanical reinforcement induced by *Chrysopogon zizanioides* (L.) Roberty roots in seven contrasting Andean soil matrices classified as CL-ML, MH, CH, OH, OL, CL, and ML. Bare-soil and Vetiver-rooted specimens were compared across three depth intervals: 0.00–0.50 m, 0.50–1.50 m, and 1.50–2.00 m. Shear-strength parameters were determined through direct shear tests under controlled normal stresses of 50, 100, and 200 kPa, and the relative amplifications of apparent cohesion (Δc) and internal friction angle (Δφ) were calculated. ANOVA, Tukey HSD post hoc tests, effect-size analysis, descriptive depth-dependent regression analyses, and shear-resistance sensitivity analysis were then applied to identify soil-dependent reinforcement patterns. The results showed that the mechanical contribution of Vetiver roots was primarily controlled by soil type. The highest relative cohesion amplification was obtained in OL soil, where Δc reached 100.0%, whereas the strongest friction-angle response was observed in CH soil, with Δφ reaching 20.1%. MH soil exhibited an intermediate and technically balanced response, combining moderate cohesion gains with relevant frictional improvement. One-way ANOVA screening indicated that soil type was the main differentiating factor controlling the Vetiver-induced response, while depth had a less dominant effect. Tukey HSD comparisons confirmed that OL exhibited a distinct cohesion-amplification pattern relative to the other soil matrices. These findings indicate that *C. zizanioides* root reinforcement should not be interpreted as a uniform plant effect but as a soil-matrix-dependent biomechanical response governed by root–soil interaction. The initial mechanical condition of the soil, rather than root content alone, determines whether Vetiver reinforcement is expressed primarily as a cohesion gain, a friction-angle gain, or a combined response. Because the statistical evidence is screening-level owing to the absence of replication within soil–depth cells, these patterns should support soil-specific interpretation rather than be applied as universal amplification factors.

## 1. Introduction

Plant-based bioengineering has gained increasing relevance as an environmentally compatible strategy for improving the behaviour of soils exposed to erosion, degradation, and near-surface instability. Unlike conventional solutions based exclusively on rigid or inert elements, ecological engineering approaches incorporate living organisms as functional components capable of modifying the physical conditions of the medium in which they grow [1,2]. In this context, vegetation should not be interpreted only as a protective surface cover but also as a biological system capable of influencing soil structure, stress transfer, and the mechanical resistance of the soil–root matrix [3,4].

Among the mechanisms associated with vegetation effects, root-induced mechanical reinforcement is one of the most relevant processes for near-surface soil stabilisation. Roots can increase shear resistance through anchorage, particle interlocking, tensile resistance mobilisation, and stress transfer across the shear zone [4,5]. These mechanisms are commonly expressed experimentally as changes in apparent shear-strength parameters, particularly apparent cohesion and, to a lesser extent, the internal friction angle [6,7]. However, the magnitude of these changes depends not only on root presence but also on root architecture, root density, reinforcement depth, moisture condition, root orientation, root diameter, root–soil interface behaviour, and the physical and mechanical properties of the surrounding soil matrix [3,8,9,10,11,12,13,14]. Experimental and analytical studies have shown that the mobilised reinforcement may vary with soil moisture, void ratio, normal stress, root concentration, and the dominant root failure mechanism, including tensile breakage, pull-out, bending, and progressive mobilisation [9,10,11,15,16,17]. Direct shear studies comparing texturally contrasting soils have shown that the same root system can increase cohesion by more than 200% in sandy soils while producing comparatively modest, more balanced cohesion and friction-angle gains in clayey soils, confirming that soil texture and plasticity regulate not only the magnitude but also the mechanism of root reinforcement [18].

*Chrysopogon zizanioides* (L.) Roberty, commonly known as Vetiver grass, is a perennial grass of the Poaceae family widely used for erosion control, degraded-soil rehabilitation, and bioengineering applications. Its dense, fibrous, and predominantly vertical root system makes it particularly relevant for applications requiring shallow and near-surface soil reinforcement. Previous studies have shown that the biomechanical properties of *Chrysopogon* roots may contribute to soil reinforcement and substrate stabilisation, although the measured response varies with soil type, root concentration, suction, root length, specimen density, water condition, and testing configuration [15,19,20,21,22,23,24,25,26]. Vetiver-specific laboratory studies have reported changes in apparent cohesion, internal friction angle, deformation behaviour, and overall shear resistance, confirming that the mechanical contribution of the species cannot be transferred between soils without considering the condition of the host matrix [15,25,26].

Despite increasing interest in Vetiver-based bioengineering, important limitations remain in the transferability of published reinforcement parameters. Most experimental studies have evaluated individual soil types, specific root concentrations, or particular hydrological conditions, whereas fewer studies have compared the response of the same species across a broad range of soil matrices under a common testing framework [11,13,15,25,26,27]. Reviews of root-reinforcement modelling also emphasise that the mechanical contribution assigned to vegetation depends on the adopted model, the spatial distribution of roots, the soil state, and the assumed root failure mechanism [16,17,27]. This limitation is particularly relevant in Andean environments, where soils may differ markedly in plasticity, organic content, texture, structure, and initial shear strength. Under these conditions, the soil–root system should be evaluated as a matrix-dependent interaction rather than as a uniform plant effect.

From an experimental perspective, direct shear testing provides a practical means of quantifying root-induced changes in the failure envelope and deriving apparent Mohr–Coulomb parameters under controlled normal stresses [10,11,15,28]. Laboratory and large-scale direct shear investigations have shown that rooted-soil behaviour may depend on root density, void ratio, soil saturation, dilatancy, imposed shear displacement, and the number and orientation of roots intersecting the shear plane [10,11,15]. The method is therefore useful for comparing bare and root-affected conditions, although the fitted values of apparent cohesion and friction angle should be interpreted as integrated parameters of the tested soil–root composite rather than as intrinsic properties of the plant species.

Within this framework, the present study evaluates the biomechanical reinforcement induced by *Chrysopogon zizanioides* roots in seven Andean soil matrices classified as CL-ML, MH, CH, OH, OL, CL, and ML. The complete experimental matrix was used to compare Vetiver-induced responses as a function of soil type and depth. Bare-soil and Vetiver-rooted specimens were evaluated across three depth intervals, and apparent cohesion and internal friction angle were derived from direct shear tests conducted under normal stresses of 50, 100, and 200 kPa. Relative amplification, effect-size analysis, screening-level ANOVA, exploratory Tukey HSD comparisons, descriptive depth-dependent regressions, and shear-resistance sensitivity analysis were then applied. After analysis of the complete dataset, OL, CH, and MH were identified a posteriori as representative response patterns associated with relative cohesion amplification, friction-angle amplification, and a combined mechanical response, respectively. Because each soil–depth combination yielded a single amplification value, the statistical analyses were interpreted as exploratory screening rather than confirmatory inference. The central hypothesis was that the mechanical contribution of Vetiver is controlled by root–soil matrix interaction and cannot be represented by a uniform reinforcement factor.

Despite this recognised dependence of root reinforcement on substrate properties, no previous Vetiver study has systematically compared a broad, USCS-representative set of Andean soil matrices under a single testing and statistical protocol, which limits the transferability of reinforcement values reported for individual soil types. To address this gap, the seven soil matrices selected here were chosen to span the range of plasticity, organic content, and initial shear strength typical of near-surface Andean soils, so that soil-dependency could be evaluated across contrasting rather than similar substrates. Because each soil–depth combination yielded a single amplification value, with no replication within cells, a one-way ANOVA screening with Tukey HSD post hoc comparisons, rather than a factorial model with an interaction term, was adopted as the statistical scheme most appropriate for a first, exploratory differentiation of soil-type and depth effects.

## 2. Materials and Methods

### 2.1. Experimental Design and Methodological Framework

This study was designed to evaluate the biomechanical contribution of *Chrysopogon zizanioides* (L.) Roberty roots to the modification of shear-strength parameters in contrasting Andean soil matrices. The methodological approach compared bare-soil specimens and Vetiver-rooted specimens across seven soil classes and three depth intervals. The complete experimental matrix included seven soils classified as CL-ML, MH, CH, OH, OL, CL, and ML. Within this global matrix, three representative response patterns were emphasised during interpretation: OL, associated with the highest relative cohesion amplification; CH, associated with the strongest internal friction-angle amplification; and MH, representing an intermediate soil with a mechanically balanced response.

**Figure 1:**
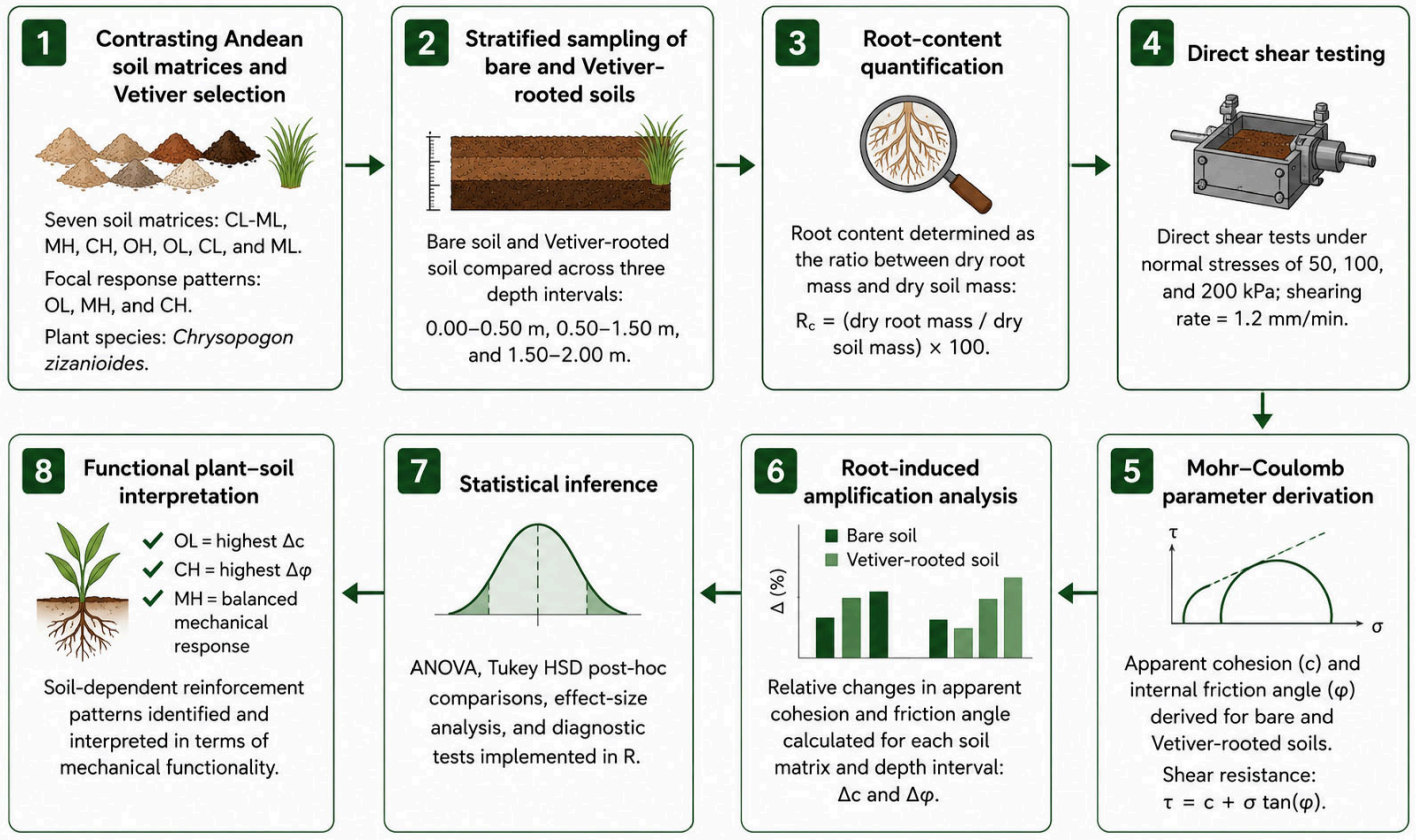
Experimental–statistical workflow for evaluating Vetiver-induced biomechanical reinforcement in Andean soils. The workflow integrates eight sequential stages: selection of contrasting Andean soil matrices and *Chrysopogon zizanioides*; stratified sampling of bare and Vetiver-rooted soils; root-content quantification; direct shear testing; derivation of Mohr–Coulomb parameters; calculation of root-induced amplification in apparent cohesion and internal friction angle; statistical inference using ANOVA, Tukey HSD, effect-size analysis and diagnostic tests; and functional interpretation of soil-dependent plant–root reinforcement patterns.

The methodological workflow used in this study is shown in Figure 1. This framework links the selection of Vetiver and contrasting soil matrices, stratified sampling, root-content quantification, direct shear testing, Mohr–Coulomb parameter derivation, root-induced amplification analysis, statistical inference, and functional plant–soil interpretation (see also Supplementary Figure S1 and Supplementary Table S1 for the complete workflow diagram and variable dictionary).

The complete experimental matrix (seven soil classes and three depth intervals) was retained throughout the amplification calculations, the one-way ANOVA/Tukey HSD screening, and the shear-resistance sensitivity analysis, so that all seven matrices contribute to the statistical evidence of soil-dependency reported in Section 3. For detailed mechanistic discussion, three matrices, OL, CH, and MH, were selected a posteriori, after the amplification results (See Section 3.4 ) identified them as the soils exhibiting, respectively, the maximum relative cohesion amplification, the maximum friction-angle amplification, and the most balanced combined response. The remaining four matrices (CL-ML, OH, CL, and ML) occupy intermediate positions along the same cohesion–friction response continuum and are therefore discussed comparatively in Section 3.4, Section 3.5 and Section 4 rather than developed into individual mechanistic subsections, while remaining fully represented in the statistical and sensitivity analyses.

### 2.2. Soil Matrices

Seven Andean soil matrices were analysed according to the Unified Soil Classification System: CL-ML, MH, CH, OH, OL, CL, and ML. These matrices represent contrasting conditions of texture, plasticity, organic content, and initial shear strength, allowing the soil dependency of Vetiver-induced reinforcement to be evaluated. For each soil matrix, the bare-soil condition was used as the mechanical reference against which Vetiver-rooted specimens were compared (full bare-soil Mohr–Coulomb parameters in Supplementary Table S3).

Before presenting the experimental factors, the study design was organised to ensure correspondence between soil type, root condition, depth interval, mechanical parameters, and statistical response variables. The complete structure of the experimental design is summarised in Table 1.

### 2.3. Plant Material, Taxonomic Identity, and Experimental Scope

The plant material considered in this study corresponded to *Chrysopogon zizanioides* (L.) Roberty, commonly known as Vetiver grass. This species belongs to the Poaceae family and was selected because of its dense, fibrous, and predominantly vertical root system, a functional trait relevant to shallow and near-surface soil reinforcement. Previous studies have shown that root architecture and root biomechanical properties influence the mechanical behaviour of soil–root systems [3,19].

The Vetiver plant material was obtained from the Municipal Nursery of Loja, Ecuador (3°57′25.01″ S, 79°13′03.27″ W), as a commercially propagated lot collected on 6 October 2025; it was therefore not derived from wild populations, protected habitats, or genetic modification or molecular work. Because the study involved no taxonomic revision, floristic inventory, or biodiversity assessment, no herbarium voucher was prepared. Taxonomic traceability instead relies on the accepted scientific name, the documented nursery source, the collection coordinates and date, and photographic records of the plant and root-containing specimens. The biological interpretation of the study was accordingly limited to root presence, root-content distribution, and their association with changes in apparent Mohr–Coulomb shear-strength parameters.

Field-scale ecological processes such as evapotranspiration, suction evolution, plant surcharge, wind loading, nutrient limitation, phytotoxicity, long-term establishment, and seasonal root development were not directly quantified. Accordingly, the results should be interpreted as experimental evidence of root-mediated biomechanical reinforcement under laboratory conditions, rather than as a complete ecological or field-scale phytostabilisation assessment.

### 2.4. Stratified Sampling of Bare and Vetiver-Rooted Soils

Sampling was organised to compare two experimental conditions: bare soil and Vetiver-rooted soil. Bare-soil specimens represented the reference condition for each soil matrix, whereas Vetiver-rooted specimens represented the soil–root condition. This comparison allowed the mechanical effect of Vetiver roots to be evaluated relative to the initial strength parameters of each soil.

To assess the influence of depth, Vetiver-rooted specimens were organised into three depth intervals: upper layer, 0.00–0.50 m; middle layer, 0.50–1.50 m; and deep layer, 1.50–2.00 m. This stratification was adopted to determine whether root-induced reinforcement varied with depth and whether this variation was consistent across soil matrices. The same soil classification and depth structure were maintained throughout the root-content, shear-strength, amplification, and statistical analyses.

### 2.5. Root-Content Quantification

Root content was quantified to describe the fraction of Vetiver roots contained in each soil–root specimen. After sample processing, roots were carefully separated from the soil matrix, cleaned to remove adhering particles, dried, and weighed. Root content was calculated before evaluating its relationship with the observed changes in shear-strength parameters.

Root content, Rc, was calculated using Equation (1):

(1) $$\mathrm{Rc}=\left(\frac{\mathrm{Mdry}\;\mathrm{roots}}{\mathrm{Mdry}\;\mathrm{soil}}\right)\times 100$$

where Rc is the root-content percentage (%), Mdry roots is the dry mass of roots, and Mdry soil is the dry mass of soil in the corresponding specimen. Rc was used as a descriptive indicator of root presence and as Supporting Information for interpreting changes in apparent cohesion and internal friction angle (complete root-content dataset in Supplementary Table S4 and Supplementary Figure S2).

### 2.6. Direct Shear Testing

Direct shear testing was used to determine the shear-strength parameters of bare and Vetiver-rooted specimens. This method is suitable for evaluating root reinforcement because it allows soil–root specimens to be sheared under controlled normal stresses and enables the resulting failure envelope to be interpreted using Mohr–Coulomb parameters [28].

Three normal stresses of 50, 100, and 200 kPa were applied during testing. A constant shearing rate of 1.2 mm/min was used for all seven matrices to maintain procedural consistency between bare-soil and Vetiver-rooted specimens. The rate was not optimised separately for each matrix or derived from soil-specific consolidation-time measurements. Consequently, the resulting apparent cohesion and friction-angle parameters are interpreted as comparative values obtained under the adopted laboratory protocol rather than as universally validated drained design parameters.

### 2.7. Mohr–Coulomb Parameter Derivation

After direct shear testing, the shear-strength response was interpreted using the Mohr–Coulomb failure criterion. The shear resistance was calculated using Equation (2):

(2) τ = c + σ tan(φ)

where τ is the shear resistance, c is the apparent cohesion, σ is the applied normal stress, and φ is the internal friction angle. For each soil matrix and depth condition, c and φ were derived from the failure envelope obtained from the direct shear results (complete Vetiver-rooted Mohr–Coulomb parameters in Supplementary Table S5, with corresponding heatmaps in Supplementary Figure S3 and failure envelopes in Supplementary Figure S9).

The parameters obtained from bare-soil specimens were used as the baseline condition, whereas the parameters obtained from Vetiver-rooted specimens represented the reinforced condition. This comparison allowed the study to determine whether Vetiver-induced reinforcement was mainly expressed as an increase in apparent cohesion, a modification of the internal friction angle, or a combined change in both parameters.

### 2.8. Root-Induced Amplification Calculation

The relative amplification induced by Vetiver roots was calculated to compare the reinforced and bare conditions within each soil matrix. This calculation was performed separately for apparent cohesion and internal friction angle.

The relative amplification of apparent cohesion, Δc, was calculated using Equation (3):

(3) $$\Delta c=\left(\frac{\mathrm{cVetiver},d\;-\;\mathrm{cBar}}{\mathrm{cBare}}\right)\times 100$$

The relative amplification of internal friction angle, Δφ, was calculated using Equation (4):

(4) $$\Delta \varphi =\left(\frac{\varphi \mathrm{Vetiver},d\;-\;\varphi \mathrm{Bare}}{\varphi \mathrm{Bare}}\right)\times 100$$

where cVetiver,d and φVetiver,d are the apparent cohesion and internal friction angle of the Vetiver-rooted soil at depth interval d, whereas cBare and φBare are the corresponding parameters of the bare soil for the same soil matrix. These amplifications were calculated for the seven soil matrices and the three Vetiver-rooted depth intervals (complete amplification matrix and per-soil summary in Supplementary Tables S6 and S7, with heatmaps and depth profiles in Supplementary Figures S4 and S10).

### 2.9. Statistical Analysis

Statistical analysis was applied to the soil–depth amplification matrix to identify soil-dependent patterns of *Chrysopogon zizanioides* (L.) Roberty-induced reinforcement. The response variables were Δc and Δφ, while the main explanatory factors were soil type and depth interval. Because the soil–depth matrix contained one observation per soil–depth combination, soil-type and depth effects were screened separately using one-way ANOVA. No interaction term was interpreted because replication within each soil–depth cell was not available. Because of this lack of within-cell replication, the ANOVA and subsequent Tukey HSD results are interpreted throughout this manuscript as screening-level evidence of soil-dependency, intended to identify differentiating patterns, rather than as confirmatory statistical inference.

Following ANOVA, Tukey HSD post hoc comparisons were applied to identify which soil matrices differed from each other in terms of root-induced amplification. This step was used to determine whether OL formed a statistically differentiated group in terms of cohesion amplification and whether CH showed a distinct pattern in friction-angle amplification. In addition to *p*-values, effect-size indicators were calculated to support the interpretation of practical significance.

Statistical diagnostics were performed to evaluate the suitability of the parametric analyses. Residual normality was assessed using the Shapiro–Wilk test [29], and variance homogeneity was evaluated using the Brown–Forsythe test [30]. Statistical analyses were implemented in R version 4.3.2, following the reproducible data-analysis environment described by Ihaka and Gentleman [31]. When assumption diagnostics indicated potential sensitivity, the interpretation was supported by effect sizes, confidence intervals, and the consistency of soil-dependent response patterns rather than by *p*-values alone (complete ANOVA screening, diagnostics, and Tukey HSD outputs in Supplementary Tables S8–S11, with supporting figures in Supplementary Figures S5, S8, S11 and S12).

### 2.10. Depth-Dependent Trend Analysis

Depth-dependent trends were evaluated to describe how *Chrysopogon zizanioides* (L.) Roberty induced changes varied across the upper, middle, and deep intervals. For each soil matrix, simple linear regressions were fitted using the representative depth of each interval as the explanatory variable and c, φ, Δc, or Δφ as response variables.

Because each soil matrix was represented by three depth intervals, these regressions were interpreted as descriptive trends rather than as general predictive models. Therefore, coefficients of determination were used to describe the internal consistency of each trend, while *p*-values were interpreted cautiously due to the limited number of depth points per soil matrix (complete regression outputs in Supplementary Table S12  and Supplementary Figure S6).

### 2.11. Sensitivity Analysis

A shear-resistance sensitivity analysis was performed to translate changes in c and φ into changes in calculated shear resistance. For each soil matrix, depth interval, and normal stress level, shear resistance was calculated using Equation (2) for both bare-soil and Vetiver-rooted conditions.

The relative change in shear resistance, Δτ, was calculated using Equation (5):

(5) $$\Delta \tau =\left(\frac{\tau \mathrm{Vetiver},d\;-\;\tau \mathrm{Bare}}{\tau \mathrm{Bare}}\right)\times 100$$

where τVetiver,d is the shear resistance of the Vetiver-rooted soil at depth interval d, and τBare is the shear resistance of the corresponding bare soil. This analysis allowed the mechanical relevance of Vetiver-induced changes in c and φ to be evaluated under the three applied normal stresses (complete sensitivity matrices in Supplementary Tables S13–S19, with heatmap and stress-dependent profiles in Supplementary Figures S7 and S13).

### 2.12. Scope, Assumptions, and Reproducibility

This study focused on the mechanical contribution of Vetiver roots contained within the tested soil specimens. Transient hydrological processes, evapotranspiration, matric suction, plant surcharge, wind effects, nutrient limitation, phytotoxicity, and temporal evolution of the root system were not directly quantified. Therefore, the results should be interpreted as laboratory-based evidence of soil–root biomechanical reinforcement and not as direct field-scale validation.

To support reproducibility, the dataset was organised as a structured matrix including soil type, root condition, depth interval, root content, apparent cohesion, internal friction angle, Δc, Δφ, and calculated shear resistance under each normal stress level. This organisation ensured direct correspondence between the methodological workflow and the subsequent results sections.

## 3. Results

### 3.1. Experimental Dataset and Baseline Properties of Bare Soil Matrices

The experimental dataset was structured to evaluate the biomechanical effect of *Chrysopogon zizanioides* roots across seven contrasting Andean soil matrices and three depth intervals. The bare-soil condition was used as the untreated reference state for each soil matrix, whereas the Vetiver-rooted condition represented the root-affected material state. This structure allowed the effect of Vetiver roots to be interpreted as a function of both soil type and depth interval.

The baseline Mohr–Coulomb parameters of the seven bare-soil matrices are presented in Table 2. These values were used as the reference condition for calculating the relative amplification of apparent cohesion, internal friction angle, and shear resistance.

### 3.2. Vetiver Root-Content Distribution Across Soil Matrices and Depth Intervals

The root-content distribution was evaluated to describe the root fraction contained in the Vetiver-rooted specimens before interpreting their mechanical response. The root-content values for Vetiver are shown in Table 3.

The root-content values indicate that Vetiver roots were concentrated mainly in the upper layer. The highest upper-layer root contents were recorded in CL-ML, MH, and CH, with values of 0.95%, 0.95%, and 0.92%, respectively. Root content decreased with depth in all soil matrices, but measurable root content remained present in the middle and deep intervals.

**Figure 2:**
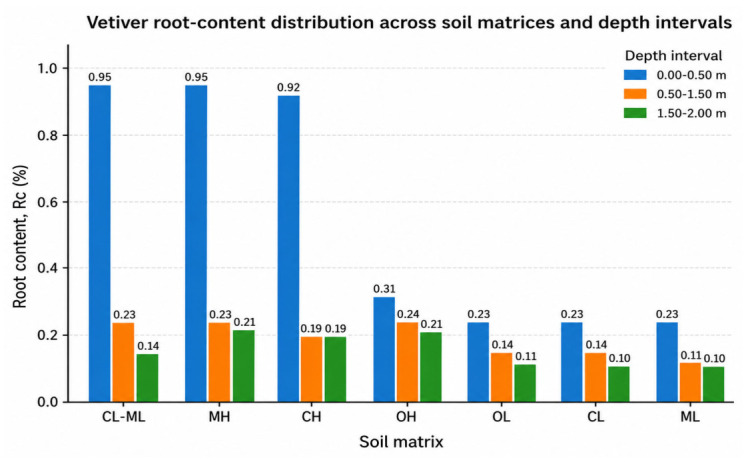
Vetiver root-content distribution across seven Andean soil matrices and three depth intervals.

Figure 2 illustrates the root-content distribution across the seven soil matrices and three depth intervals.

Root content is expressed as the percentage ratio between dry root mass and dry soil mass. The figure highlights the near-surface concentration of Vetiver roots and the reduction in root content with depth.

The highest root-content values did not necessarily correspond to the highest mechanical amplification. For example, CL-ML, MH, and CH presented the highest upper-layer root contents, whereas OL showed the highest relative cohesion amplification. This indicates that root content alone does not explain the reinforcement response and that the soil matrix plays a controlling role in the mechanical expression of Vetiver root reinforcement.

### 3.3. Vetiver-Rooted Mohr–Coulomb Shear-Strength Parameters

The direct shear results of Vetiver-rooted specimens were interpreted through the Mohr–Coulomb parameters c and φ. The resulting values are presented in Table 4 for each soil matrix and depth interval.

Vetiver-rooted specimens showed increases in apparent cohesion and internal friction angle relative to the corresponding bare-soil references. However, the magnitude of the increase varied substantially among soil matrices. The strongest absolute increase in cohesion occurred in CH, where c increased from 50.70 kPa in the bare condition to 56.01 kPa in the deep Vetiver-rooted layer. In contrast, the strongest relative cohesion response occurred in OL because of its very low bare-soil cohesion.

### 3.4. Root-Induced Amplification of Apparent Cohesion and Internal Friction Angle

The following amplification results confirm the pattern anticipated in Section 3.2 and Section 3.3: the magnitude and even the dominant component (cohesion versus friction) of Vetiver-induced reinforcement differ markedly among soil matrices. The relative amplification induced by Vetiver roots was calculated for apparent cohesion and internal friction angle using the corresponding bare-soil values as references. The calculated Δc and Δφ values are presented in Table 5.

The summary of mean and maximum amplification values by soil matrix is presented in Table 6.

**Figure 3:**
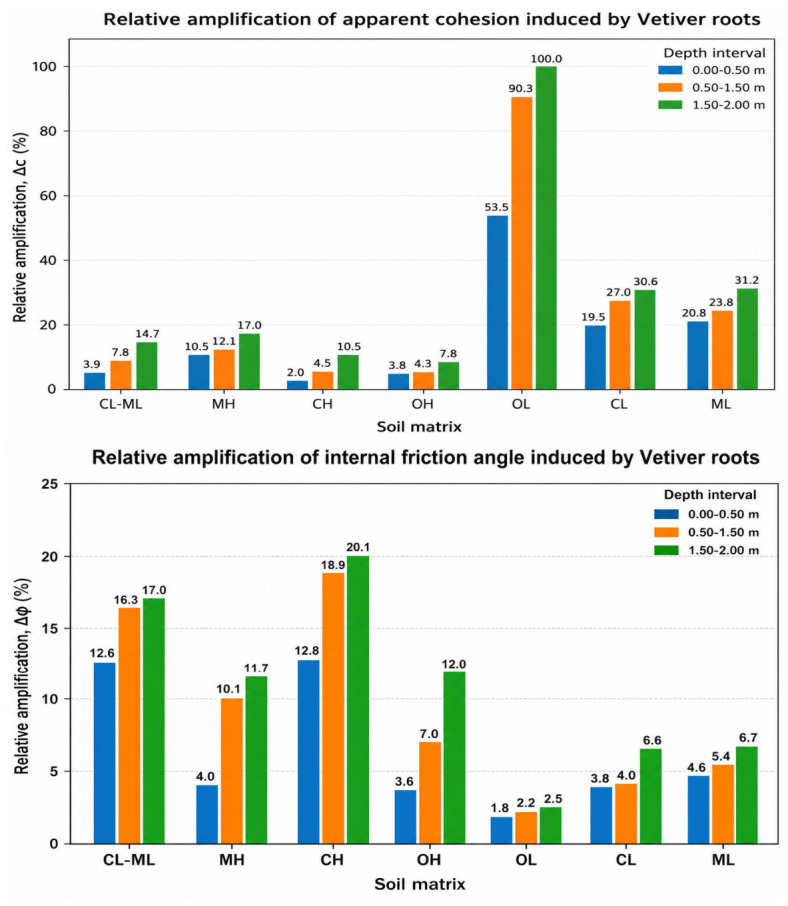
Relative amplification of apparent cohesion and internal friction angle induced by Vetiver roots. Values are expressed as percentage changes relative to the corresponding bare-soil condition for each soil matrix and depth interval.

Figure 3 compares the relative amplification of c and φ induced by Vetiver roots.

The amplification results show that Vetiver-induced reinforcement was not uniform across soil matrices. OL showed the highest relative cohesion amplification, reaching Δc = 100.0% in the deep layer. However, this result must be interpreted together with the low initial cohesion of OL. CH showed the highest friction-angle amplification, reaching Δφ = 20.1% in the deep layer. MH showed a more balanced mechanical response, with moderate cohesion amplification and relevant friction-angle improvement.

### 3.5. Statistical Evidence of Soil-Dependent Vetiver Reinforcement

At a screening level, the statistical analysis showed stronger differentiation among soil matrices than among depth intervals for both Δc and Δφ. The statistical analysis was applied to the 21 soil–depth amplification values obtained from the seven soil matrices and three depth intervals. Because the dataset contained one value for each soil–depth combination, the soil-type and depth effects were screened separately using one-way ANOVA. This approach avoids overinterpreting an interaction term that cannot be robustly estimated without replication within each soil–depth cell.

The ANOVA results are presented in Table 7. The analysis produced substantially larger effect-size estimates for soil type than for depth interval for both Δc and Δφ.

The statistical diagnostic results are summarised in Table 8. These diagnostics were used to evaluate the robustness of the ANOVA interpretation.

After ANOVA, Tukey HSD post hoc comparisons were applied to identify which soil matrices differed significantly. The significant contrasts for Δc are shown in Table 9.

The exploratory Tukey HSD comparisons identified OL as a differentiated group in terms of relative cohesion amplification. This was the strongest post hoc pattern in the dataset and supports the interpretation that Vetiver roots produced a distinct relative cohesion response in the low-cohesion OL matrix.

The significant Tukey HSD comparisons for Δφ are presented in Table 10.

The post hoc comparisons for Δφ showed that CH had a significantly stronger friction-angle response than CL, MH, ML, OH, and OL. CL-ML also showed a significant friction-angle response relative to CL, ML, and OL. These findings indicate that Vetiver-induced changes in φ were also soil-matrix-dependent, although the dominant matrix was CH rather than OL.

**Figure 4:**
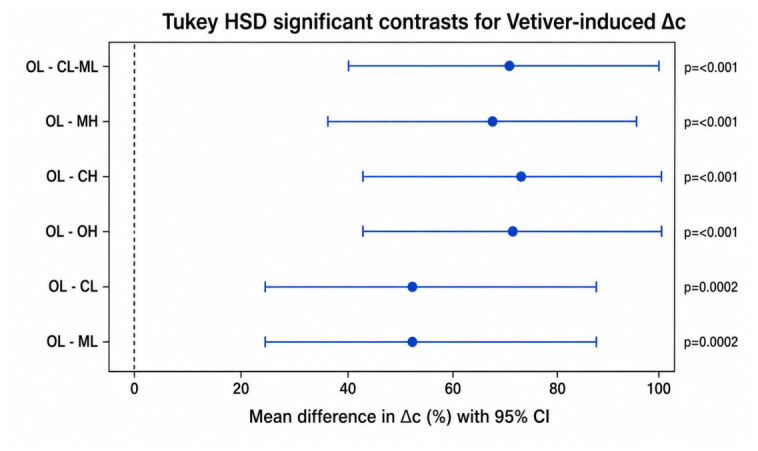

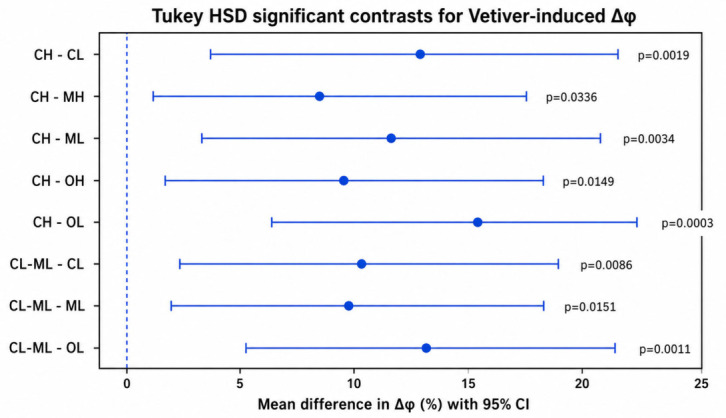
Exploratory Tukey HSD comparisons of Vetiver-induced amplification among soil matrices: significant contrasts for Δc and Δφ. Points represent mean differences, and horizontal lines represent 95% confidence intervals.

Figure 4 summarises the post hoc comparison patterns for Δc and Δφ.

Tukey HSD comparisons identify OL as the differentiated group for apparent cohesion amplification and CH as the dominant matrix for friction-angle amplification.

### 3.6. Depth-Dependent Descriptive Trends in Focal Soil Matrices

Depth-dependent trends were evaluated for the three focal soil matrices: OL, MH, and CH. These soils were selected because they represent the three main functional response patterns identified in the dataset: maximum relative cohesion amplification, balanced response, and maximum friction-angle amplification. Because each trend is fitted to only three depth points and, as shown by the ANOVA screening in Table 7, depth interval was not a statistically significant isolated factor, the following regressions are presented strictly as descriptive trends and should not be read as evidence of a depth-controlled reinforcement mechanism.

The fitted linear trends for c and φ are presented in Table 11. The representative depths used for the regressions were the midpoints of the three depth intervals: 0.25 m, 1.00 m, and 1.75 m.

### 3.7. Shear-Resistance Sensitivity to Vetiver-Modified Parameters

This final analysis tests whether the soil-dependent patterns in Δc and Δφ translate into a proportionally soil-dependent gain in practical shear resistance or whether some soils convert a large parameter change into only a modest mechanical benefit. The shear-resistance sensitivity analysis translated the Vetiver-induced changes in c and φ into changes in calculated shear resistance, τ. The analysis was performed under the same normal stress levels used in the direct shear tests: 50, 100, and 200 kPa. The mean relative change in shear resistance, Δτ, is presented in Table 12.

**Figure 5:**
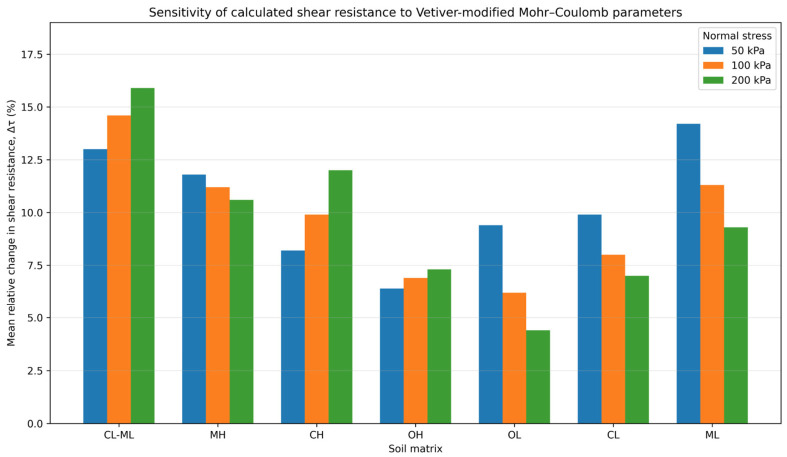
Sensitivity of calculated shear resistance to Vetiver-modified Mohr–Coulomb parameters.

Figure 5 compares the relative change in shear resistance under the three normal stress levels.

The figure compares Δτ under normal stresses of 50, 100, and 200 kPa for each soil matrix.

The sensitivity results show that the highest relative cohesion amplification did not automatically produce the highest calculated shear-resistance improvement. Although OL reached Δc = 100.0%, its mean global Δτ was 6.7%. This occurred because its friction-angle amplification was small and because the frictional component becomes increasingly important as normal stress increases. In contrast, CL-ML showed the highest mean global Δτ, reaching 14.5%, because its response involved a stronger combined contribution of cohesion and friction-angle changes. This confirms that the mechanical relevance of Vetiver reinforcement must be interpreted through the combined Mohr–Coulomb response rather than through Δc alone.

### 3.8. Integrated Functional Plant–Soil Response Patterns

The results identify three functional response patterns of Vetiver-induced reinforcement in Andean soil matrices. First, OL represented the strongest relative cohesion-amplification pattern. In this soil, c increased from 2.69 kPa in the bare condition to 5.38 kPa in the deep Vetiver-rooted layer, producing Δc = 100.0%. However, this response reflects a strong relative effect in a low-cohesion matrix, not necessarily the largest absolute mechanical gain.

Second, CH represented the strongest friction-angle-amplification pattern. In this soil, φ increased from 14.57° in the bare condition to 17.50° in the deep Vetiver-rooted layer, producing Δφ = 20.1%. This response suggests that the interaction between Vetiver roots and the clayey matrix modified not only the apparent bonding component but also the frictional component of the shear-strength envelope.

Third, MH represented a mechanically balanced response. In this soil, c increased from 31.07 kPa to 36.34 kPa, while φ increased from 21.48° to 24.00° in the deep Vetiver-rooted layer. Although MH did not show the maximum value of either Δc or Δφ, it combined moderate cohesion amplification with relevant friction-angle improvement, making it an important representative matrix for interpreting practical Vetiver-based bioengineering applications in Andean soils.

Overall, the results support the central hypothesis of this study: the biomechanical contribution of *Chrysopogon zizanioides* roots is not a uniform plant effect but a soil-matrix-dependent response. Vetiver reinforcement was expressed differently according to the baseline mechanical condition of each soil matrix. OL showed the strongest relative cohesion response, CH showed the strongest friction-angle response, and MH showed a balanced mechanical response. Therefore, the functional performance of Vetiver roots should be interpreted through the combined interaction among root content, soil matrix, apparent cohesion, internal friction angle, and shear-resistance sensitivity (see Supplementary Table S15 for the complete supplementary figure inventory, Supplementary Table S20 for the quality-control checklist, and Supplementary Table S21 for the full crosswalk between this manuscript and the Supplementary Materials).

## 4. Discussion

### 4.1. Soil Matrix as the Main Control of Vetiver-Induced Reinforcement

The results demonstrate that the biomechanical reinforcement induced by *Chrysopogon zizanioides* was not expressed as a uniform effect across the seven Andean soil matrices. Instead, the response was primarily controlled by soil type, confirming that the mechanical efficiency of Vetiver depends on root–soil matrix interaction rather than on root presence alone.

This behaviour is consistent with previous experimental and modelling studies showing that root reinforcement depends on the interaction among soil moisture, soil density, void ratio, texture, normal stress, root architecture, root concentration, diameter, orientation, tensile properties, and root–soil interface behaviour [3,4,5,9,10,11,12,13,14,15,18,26]. Large-scale direct shear tests have further shown that the peak response of root-permeated soils is related not only to root mass but also to dilatancy, void ratio, and the mechanical condition of the host soil [11]. Therefore, the differences observed among OL, CH, MH, and the remaining matrices are consistent with a composite response in which identical plant material may mobilise differently according to the initial state of the surrounding soil.

### 4.2. Cohesion-Dominated Response in OL Soil

OL soil showed the highest relative cohesion amplification, reaching Δc = 100.0% as cohesion increased from 2.69 kPa (bare) to 5.38 kPa (deep Vetiver-rooted layer). The two figures should always be read together: the absolute gain of 2.69 kPa is modest in engineering terms, and it is precisely because OL’s baseline cohesion is so low that this modest absolute change produces the largest percentage amplification in the dataset. This result therefore indicates that Vetiver roots were effective in modifying the apparent cohesion component of a low-strength organic matrix, but it should not be read as evidence that OL becomes the strongest reinforced soil overall.

Mechanistically, the response of OL is consistent with a cohesion-dominated modification of the fitted failure envelope. Root interlocking, root–soil interface resistance, and suction-related effects may have contributed to the measured increase in apparent cohesion. However, because root orientation, interface strength, matric suction, and particle rearrangement were not independently measured, the dominant root-scale mechanism cannot be identified from the present dataset. The frictional component changed only slightly, with Δφ remaining approximately 2%. This pattern agrees with approaches in which root reinforcement is represented primarily as an increase in apparent or additional cohesion [6,7]. Therefore, OL should not be interpreted as the best soil in absolute terms but rather as the matrix where Vetiver produced the most differentiated relative cohesion response, and one where the practical benefit of Vetiver should be assessed from the absolute τ gain reported in Section 3.7 rather than from Δc alone.

This interpretation is also consistent with studies showing that root concentration and root–soil interface bonding can increase the intercept of a fitted failure envelope without necessarily producing an equivalent increase in its frictional component [12,15,25]. Interface-based models further indicate that root area ratio, inclination, root diameter, embedded length, and interface cohesion and friction influence the ultimate resistance mobilised by rooted soil [12]. Accordingly, the cohesion-dominated response observed in OL should be interpreted as one possible matrix-specific expression of reinforcement rather than as a universal Vetiver mechanism.

### 4.3. Friction-Angle Response in CH Soil

In contrast to OL, CH soil showed the highest internal friction-angle amplification, with Δφ = 20.1%. This result is relevant because root reinforcement is commonly interpreted as a cohesion-dominated mechanism, whereas changes in φ are often smaller or less consistent. The response observed in CH suggests that, in high-plasticity clay, Vetiver roots may influence not only apparent cohesion but also the frictional and structural arrangement of the soil–root matrix.

This behaviour may be associated with anchorage, tensile resistance mobilisation, and stress transfer across the shear plane [4,5,28]. Unlike OL, CH is a high-plasticity clay in which particle-scale bonding is already strong, so additional apparent cohesion from roots has comparatively little room to develop (Δc = 10.5% at most); instead, root fibres crossing the shear plane are more likely to be mobilised in tension and to resist rotation and dilation of the surrounding clay aggregates as shearing proceeds, which is expressed mechanically as an increase in the mobilised friction angle rather than in cohesion. This interpretation is consistent with the DRAM analytical framework [28], in which root reinforcement is modelled as a displacement-dependent combination of root elongation, breakage, and slippage rather than as a fixed cohesion increment; in the DRAM formulation, root orientation relative to the shear plane and progressive root mobilisation with shear displacement can shift the balance of reinforcement toward the frictional component, precisely the pattern observed in CH. This makes CH a key matrix for discussing that Vetiver-induced reinforcement is not limited to cohesion enhancement but may also alter the frictional component of mechanical behaviour.

The distinction between cohesion-dominated and friction-related responses is also relevant when comparing analytical root-reinforcement models. Fibre-break and fibre-bundle formulations distribute load according to root strength and progressive failure, whereas pull-out and bending-based approaches additionally incorporate root–soil interface resistance, root stiffness, embedded length, and soil confinement [12,14,16,17]. Comparative studies have demonstrated that the selected formulation may considerably affect the predicted root contribution when the assumed failure mechanism does not match the actual root response [16]. The fitted increase in φ observed in CH may therefore represent stress-dependent composite behaviour that is not fully captured by models based exclusively on a constant additional-cohesion term.

### 4.4. MH as a Balanced Biomechanical Response

MH soil did not show the maximum Δc or Δφ values, but it exhibited a balanced response, combining moderate cohesion increases with relevant friction-angle improvement. This result is important because MH was not governed by an extreme condition, such as the very low initial cohesion of OL or the strong frictional response of CH.

From a plant-based bioengineering perspective, MH represents a practical and technically stable matrix. In Andean silty soils, a balanced response may be more useful than an extreme percentage amplification because it combines improvements in both components of shear strength. Therefore, MH represents a relevant case for interpreting Vetiver applications in soil matrices where an integrated mechanical improvement is required.

### 4.5. Root Content Alone Does Not Explain the Mechanical Response

This agrees with previous experimental–numerical studies reporting that increases in mechanical performance cannot be attributed exclusively to root biomass but rather to the interaction between root architecture and the surrounding soil matrix [23].

The root-content results showed that Vetiver had the highest upper-layer root concentrations in CL-ML, MH, and CH. However, the highest relative cohesion amplification occurred in OL, where root content was lower. This demonstrates that root percentage alone does not explain the reinforcement effect.

This result agrees with studies showing that root mass or root-content percentage cannot fully describe mechanical reinforcement when root diameter, orientation, area ratio, embedded length, branching, tensile properties, and interface bonding are not considered [12,13,14]. In particular, differences in root size and orientation can modify stiffness, transverse resistance, the area of mobilised soil, and the likelihood of pull-out or breakage [12,14]. Root content should therefore be treated as a descriptive indicator of root presence rather than as a complete mechanical predictor.

This finding is important for a plant-centred interpretation. The fibrous root architecture of Vetiver provides reinforcement potential, but the mechanical expression of that potential depends on the matrix in which the roots interact. In other words, it is not sufficient to measure how much root material is present; it is also necessary to interpret how that material interacts with soil texture, plasticity, structure, organic content, and initial shear strength. Studies on functional root traits and root-reinforced soils similarly emphasize that mechanical effects must be evaluated together with soil properties rather than as isolated biological variables [3,19].

### 4.6. Statistical Evidence of a Soil-Dependent Response

The screening-level statistical analysis, consistent with the absence of within-cell replication noted in Section 2.9, confirmed that soil type was the main differentiating factor in the Vetiver-induced response. Tukey HSD comparisons identified OL as a differentiated matrix for cohesion amplification, whereas CH stood out for friction-angle amplification (complete pairwise contrasts in Supplementary Tables S10 and S11, and Supplementary Figure S12). These results support the interpretation that Vetiver does not produce homogeneous reinforcement but rather a soil-matrix-dependent biomechanical response.

This finding agrees with recent reviews and comparative modelling studies indicating that vegetation-based stabilisation is highly context-dependent and should not be generalised without considering soil type, root distribution, hydrological condition, spatial variability, root failure mechanism, and mechanical boundary conditions [16,17,27,32,33]. Root-reinforcement reviews also emphasise that uncertainty in the spatial distribution and mobilisation of roots may be as important as uncertainty in conventional parameters such as cohesion, friction angle, and hydraulic conductivity [27].

### 4.7. Implications for Plant-Based Bioengineering in Andean Soils

The results have direct implications for the use of Vetiver as a bioengineering species in Andean environments. In low-cohesion organic soils such as OL, Vetiver may be useful for increasing apparent cohesion. In high-plasticity clays such as CH, its contribution may be more related to modification of the frictional component. In elastic silts such as MH, Vetiver may provide a more balanced improvement in shear-strength parameters.

Therefore, Vetiver should not be recommended using a generic reinforcement value. Recent experimental evidence has likewise emphasised that the mechanical benefits provided by Vetiver are controlled by the combined response of roots and substrate properties, reinforcing the need for soil-specific design criteria in bioengineering applications [23]. This recommendation is also consistent with recent experimental evidence showing that vegetation-based reinforcement should be incorporated into geotechnical design using site-specific mechanical parameters rather than generalised reinforcement assumptions [34].

This soil-specific recommendation is consistent with Vetiver studies conducted in clayey sand, cohesionless soil, and silty-clayey engineering materials, where changes in apparent cohesion, friction angle, density, moisture-related behaviour, and overall shear resistance varied with root concentration and specimen condition [15,25,26]. Consequently, parameters obtained for Vetiver in one substrate should not be transferred directly to another without laboratory or field calibration.

### 4.8. Limitations and Future Research

This study focused on the mechanical contribution of Vetiver roots contained within direct shear specimens. Hydrological processes such as evapotranspiration, matric suction, rainfall interception, and seasonal water-content variation were not quantified. Root tensile strength, root diameter distribution, temporal evolution of the root system, and field-scale ecological establishment were also outside the scope of this laboratory-based analysis.

Future studies should integrate root tensile testing, root diameter classification, suction-controlled shear testing, and long-term field monitoring. Coupled hydro-mechanical models would also be useful for evaluating how Vetiver roots interact with rainfall infiltration and transient pore-water pressure conditions. These extensions would allow *Chrysopogon zizanioides* to be interpreted within a more complete plant–soil–water system.

## 5. Conclusions

This study showed that the mechanical response associated with *Chrysopogon zizanioides* varied more strongly among soil matrices than could be explained by root-content percentage alone. Vetiver increased shear-strength parameters in all seven matrices tested, but the magnitude and, more importantly, the dominant mechanism of reinforcement, cohesion gain, friction-angle gain, or a balanced combination of both were governed by the initial mechanical condition of each soil rather than by root presence alone.

In the low-cohesion OL matrix, Vetiver roots produced the largest relative cohesion amplification (Δc = 100.0%, from 2.69 to 5.38 kPa). Because the baseline cohesion was low, this result should be interpreted together with the corresponding absolute increase of 2.69 kPa. The response is consistent with a cohesion-dominated modification of the fitted failure envelope, although the responsible root-scale mechanism was not measured directly.

In the high-plasticity CH matrix, Vetiver roots produced the largest relative amplification of the fitted internal friction angle (Δφ = 20.1%). This result is consistent with a stress-dependent composite response involving root mobilisation and soil–root interaction; however, it does not independently demonstrate that anchorage or tensile mobilisation was the dominant root-scale mechanism. Soil-specific apparent shear-strength parameters should therefore be used when evaluating this response in stability calculations.

In elastic silts such as MH, Vetiver produced a balanced combination of moderate cohesion and friction-angle gains, without reaching the extreme of either OL or CH; this response pattern makes MH-type soils a favourable target for integrated, dual-parameter Vetiver-based bioengineering designs where neither cohesion nor friction can be neglected.

Across all seven matrices, root content alone did not explain the magnitude of the mechanical response: the soils with the highest upper-layer root content (CL-ML, MH, and CH) were not those with the highest amplification (OL), confirming that soil properties, not root biomass, constitute the primary control on the biomechanical expression of Vetiver reinforcement. Because the statistical evidence supporting these patterns is screening-level, given the absence of replication within soil–depth cells, the three soil-specific mechanisms identified here should be treated as a basis for soil-specific Vetiver design rather than as fixed universal amplification factors.

Overall, the findings provide experimental evidence that *Chrysopogon zizanioides* behaves as a soil-dependent biomechanical reinforcement species. This perspective contributes to the understanding of plant–soil interactions in bioengineering applications and provides useful information for the design of vegetation-based stabilisation strategies in Andean environments.

## Figures and Tables

**Table 1:** Experimental factors considered in the assessment of Vetiver-induced soil reinforcement.

Factor	Levels or Variables Considered
Plant species	*Chrysopogon zizanioides*
Soil condition	Bare soil; Vetiver-rooted soil
Soil matrix	CL-ML, MH, CH, OH, OL, CL, ML
Depth interval	0.00–0.50 m; 0.50–1.50 m; 1.50–2.00 m
Mechanical parameters	Apparent cohesion, c; internal friction angle, φ
Derived response variables	Δc; Δφ; Δτ
Statistical factors	Soil type; depth interval

**Table 2:** Bare-soil Mohr–Coulomb parameters used as reference conditions for Vetiver-induced amplification analysis.

Soil Matrix	Apparent Cohesion, c (kPa)	Internal Friction Angle, φ (°)	Interpretative Role in This Study
CL-ML	25.77	24.34	Mixed fine-grained matrix
MH	31.07	21.48	Focal balanced-response matrix
CH	50.70	14.57	Focal high-cohesion clayey matrix
OH	18.53	14.87	Organic high-plasticity matrix
OL	2.69	29.49	Focal low-cohesion organic matrix
CL	6.82	27.82	Low-cohesion clayey matrix
ML	16.96	26.10	Silty matrix with moderate baseline strength

The baseline parameters show marked differences among the seven soils. CH had the highest initial cohesion, whereas OL and CL showed the lowest cohesion values. In contrast, OL, CL, and ML showed the highest friction angles. This contrast is important because relative cohesion amplification may become very large in low-cohesion soils, even when the absolute increase in cohesion remains moderate. Therefore, both relative and absolute changes were considered when interpreting Vetiver-induced reinforcement.

**Table 3:** Vetiver root-content distribution by soil matrix and depth interval.

Soil Matrix	Rc Upper Layer, 0.00–0.50 m (%)	Rc Middle Layer, 0.50–1.50 m (%)	Rc Deep Layer, 1.50–2.00 m (%)	Mean Rc (%)
CL-ML	0.95	0.23	0.14	0.44
MH	0.95	0.23	0.21	0.46
CH	0.92	0.19	0.19	0.43
OH	0.31	0.22	0.21	0.25
OL	0.23	0.14	0.11	0.16
CL	0.23	0.14	0.10	0.16
ML	0.23	0.11	0.10	0.15

**Table 4:** Vetiver-rooted Mohr–Coulomb shear-strength parameters by soil matrix and depth interval.

Soil Matrix	Depth Interval	c Vetiver (kPa)	φ Vetiver (°)
CL-ML	0.00–0.50 m	26.78	27.41
CL-ML	0.50–1.50 m	27.78	28.31
CL-ML	1.50–2.00 m	29.55	28.47
MH	0.00–0.50 m	34.33	22.33
MH	0.50–1.50 m	34.83	23.65
MH	1.50–2.00 m	36.34	24.00
CH	0.00–0.50 m	51.70	16.43
CH	0.50–1.50 m	52.96	17.33
CH	1.50–2.00 m	56.01	17.50
OH	0.00–0.50 m	19.23	15.40
OH	0.50–1.50 m	19.33	15.91
OH	1.50–2.00 m	19.98	16.66
OL	0.00–0.50 m	4.13	30.01
OL	0.50–1.50 m	5.12	30.13
OL	1.50–2.00 m	5.38	30.22
CL	0.00–0.50 m	8.15	28.88
CL	0.50–1.50 m	8.66	28.94
CL	1.50–2.00 m	8.91	29.67
ML	0.00–0.50 m	20.49	27.31
ML	0.50–1.50 m	21.00	27.50
ML	1.50–2.00 m	22.25	27.85

**Table 5:** Relative amplification of apparent cohesion and internal friction angle induced by Vetiver roots.

Soil Matrix	Depth Interval	Δc (%)	Δφ (%)
CL-ML	0.00–0.50 m	3.9	12.6
CL-ML	0.50–1.50 m	7.8	16.3
CL-ML	1.50–2.00 m	14.7	17.0
MH	0.00–0.50 m	10.5	4.0
MH	0.50–1.50 m	12.1	10.1
MH	1.50–2.00 m	17.0	11.7
CH	0.00–0.50 m	2.0	12.8
CH	0.50–1.50 m	4.5	18.9
CH	1.50–2.00 m	10.5	20.1
OH	0.00–0.50 m	3.8	3.6
OH	0.50–1.50 m	4.3	7.0
OH	1.50–2.00 m	7.8	12.0
OL	0.00–0.50 m	53.5	1.8
OL	0.50–1.50 m	90.3	2.2
OL	1.50–2.00 m	100.0	2.5
CL	0.00–0.50 m	19.5	3.8
CL	0.50–1.50 m	27.0	4.0
CL	1.50–2.00 m	30.6	6.6
ML	0.00–0.50 m	20.8	4.6
ML	0.50–1.50 m	23.8	5.4
ML	1.50–2.00 m	31.2	6.7

**Table 6:** Summary of Vetiver-induced amplification by soil matrix.

Soil Matrix	Mean Δc (%)	Maximum Δc (%)	Mean Δφ (%)	Maximum Δφ (%)	Main Response Pattern
CL-ML	8.8	14.7	15.3	17.0	High friction-angle response
MH	13.2	17.0	8.6	11.7	Balanced cohesion–friction response
CH	5.7	10.5	17.3	20.1	Highest friction-angle amplification
OH	5.3	7.8	7.5	12.0	Moderate response
OL	81.3	100.0	2.2	2.5	Highest relative cohesion amplification
CL	25.7	30.6	4.8	6.6	Moderate-to-high cohesion response
ML	25.3	31.2	5.6	6.7	Moderate-to-high cohesion response

**Table 7:** ANOVA screening of Vetiver-induced amplification by soil type and depth interval.

Response Variable	Factor Screened	F-Value	*p*-Value	η^2^	Interpretation
Δc	Soil type	20.80	3.18 × 10^−6^	0.899	Strong soil-matrix effect
Δc	Depth interval	0.45	0.644	0.048	Not significant as an isolated factor
Δφ	Soil type	10.90	1.36 × 10^−4^	0.824	Strong soil-matrix effect
Δφ	Depth interval	1.24	0.314	0.121	Not significant as an isolated factor

The ANOVA results indicate that the type of soil matrix controlled the Vetiver-induced amplification response more strongly than depth alone. Soil type explained 89.9% of the variability in Δc and 82.4% of the variability in Δφ. In contrast, depth interval alone did not show a statistically significant isolated effect for either response variable. Therefore, the reinforcement pattern should be interpreted primarily as soil-matrix-dependent.

**Table 8:** Diagnostic tests for the ANOVA screening models.

Response Variable	Model Screened	Shapiro–Wilk *p*-Value	Brown–Forsythe *p*-Value	Diagnostic Interpretation
Δc	Soil type	0.003	0.375	Residual normality not satisfied; variance homogeneity not rejected
Δc	Depth interval	<0.001	0.864	Residual normality not satisfied; variance homogeneity not rejected
Δφ	Soil type	0.442	0.689	Diagnostics acceptable
Δφ	Depth interval	0.084	0.622	Diagnostics acceptable

For Δc, the residual normality assumption was not satisfied, mainly because OL behaved as an extreme cohesion-amplification group. However, variance homogeneity was not rejected. Therefore, the interpretation of Δc was not based only on the *p*-value but also on the large effect size, the consistency of the OL response across the three depth intervals, and the post hoc comparison results. For Δφ, the diagnostic tests supported the use of the ANOVA screening more directly.

**Table 9:** Significant Tukey HSD post hoc comparisons for Vetiver-induced cohesion amplification, Δc.

Contrast	Mean Difference (%)	95% Confidence Interval (%)	Adjusted *p*-Value	Result
OL − CL-ML	72.49	44.08 to 100.90	<0.001	Significant
OL − MH	68.10	39.69 to 96.51	<0.001	Significant
OL − CH	75.65	47.24 to 104.06	<0.001	Significant
OL − OH	75.98	47.57 to 104.39	<0.001	Significant
OL − CL	55.58	27.17 to 83.99	0.0002	Significant
OL − ML	56.01	27.60 to 84.42	0.0002	Significant

**Table 10:** Significant Tukey HSD post hoc comparisons for Vetiver-induced internal friction-angle amplification, Δφ.

Contrast	Mean Difference (%)	95% Confidence Interval (%)	Adjusted *p*-Value	Result
CH − CL	12.44	4.30 to 20.59	0.0019	Significant
CH − MH	8.68	0.53 to 16.82	0.0336	Significant
CH − ML	11.70	3.56 to 19.85	0.0034	Significant
CH − OH	9.74	1.59 to 17.89	0.0149	Significant
CH − OL	15.14	6.99 to 23.28	0.0003	Significant
CL-ML − CL	10.47	2.32 to 18.62	0.0086	Significant
CL-ML − ML	9.73	1.58 to 17.88	0.0151	Significant
CL-ML − OL	13.16	5.01 to 21.31	0.0011	Significant

**Table 11:** Descriptive depth-dependent trends for focal soil matrices.

Soil Matrix	Parameter	Linear Trend	R^2^	*p*-Value	Interpretation
OL	c	c(z) = 4.043 + 0.833z	0.898	0.207	Increasing cohesion trend
OL	φ	φ(z) = 29.980 + 0.140z	0.993	0.052	Nearly linear but small absolute change
MH	c	c(z) = 33.827 + 1.340z	0.922	0.180	Increasing cohesion trend
MH	φ	φ(z) = 22.213 + 1.113z	0.899	0.206	Increasing friction-angle trend
CH	c	c(z) = 50.683 + 2.873z	0.946	0.150	Increasing cohesion trend
CH	φ	φ(z) = 16.373 + 0.713z	0.866	0.239	Increasing friction-angle trend

The regression results showed internally consistent increasing trends with depth for the focal matrices. However, because each soil matrix was represented by only three depth intervals, these regressions were interpreted as descriptive trends rather than as predictive models. The high R^2^ values indicate consistent within-soil patterns, but the *p*-values did not reach formal statistical significance because of the limited degrees of freedom. Therefore, the depth trends should be used to support interpretation, not as independent proof of a depth-controlled mechanism.

**Table 12:** Mean relative variation in calculated shear resistance induced by Vetiver-modified parameters.

Soil Matrix	Mean Δτ at 50 kPa (%)	Mean Δτ at 100 kPa (%)	Mean Δτ at 200 kPa (%)	Mean Global Δτ (%)	Sensitivity Interpretation
CL-ML	13.0	14.6	15.9	14.5	Highest overall shear-resistance sensitivity
MH	11.8	11.2	10.6	11.2	Balanced improvement
CH	8.2	9.9	12.0	10.1	Friction-angle-driven improvement
OH	6.4	6.9	7.3	6.8	Moderate response
OL	9.4	6.2	4.4	6.7	High Δc but moderate Δτ response
CL	9.9	8.0	7.0	8.3	Moderate cohesion-driven response
ML	14.2	11.3	9.3	11.6	Higher sensitivity at low normal stress

## Data Availability

The original data presented in this study are openly available in Zenodo at https://doi.org/10.5281/zenodo.21011902. The repository contains the complete experimental dataset, including the geotechnical characterization of seven representative Andean soil classes, direct shear test results for bare and *Chrysopogon zizanioides*-rooted specimens, Mohr–Coulomb shear strength parameters, root-content measurements, root-induced amplification calculations (Δc and Δφ), statistical analyses (ANOVA, Tukey HSD, and effect size), and shear-resistance sensitivity analyses supporting the findings reported in this article.

## Supplementary Materials

The following supporting information can be downloaded at: https://www.mdpi.com/article/10.3390/plants15152328/s1, Figure S1. Experimental–statistical workflow. Figure S2. Vetiver root-content heatmap. Figure S3. Vetiver-rooted Mohr–Coulomb parameter heatmaps. Figure S4. Amplification heatmaps for Δc and Δφ. Figure S5. Soil-matrix group means and 95% confidence intervals. Figure S6. Depth-dependent trends in focal soil matrices. Figure S7. Mean shear-resistance sensitivity heatmap. Figure S8. Diagnostic plots for soil-type ANOVA models. Figure S9. Mohr–Coulomb failure envelopes for bare and Vetiver-rooted conditions. Figure S10. Depth profiles of Δc and Δφ. Figure S11. Effect sizes for ANOVA screening models. Figure S12. Forest plots of significant Tukey HSD contrasts. Figure S13. Stress-dependent shear-resistance sensitivity profiles. Table S1. Experimental variables and analytical role. Table S2. Minimum variable dictionary for reproducing the analysis. Table S3. Bare-soil Mohr–Coulomb parameters used as reference conditions. Table S4. Vetiver root-content distribution by soil matrix and depth interval. Table S5. Vetiver-rooted Mohr–Coulomb parameters by soil matrix and depth interval. Table S6. Complete Vetiver-induced amplification matrix for apparent cohesion and internal friction angle. Table S7. Summary of Vetiver-induced amplification by soil matrix. Table S8. One-way ANOVA screening results. Table S9. Diagnostic tests for the one-way ANOVA screening models. Table S10. Complete Tukey HSD pairwise comparisons for Δc. Table S11. Complete Tukey HSD pairwise comparisons for Δφ. Table S12. Descriptive depth-dependent trends for focal soil matrices. Table S13. Complete shear-resistance sensitivity matrix. Table S14. Mean shear-resistance sensitivity by soil matrix and normal stress. Table S15. Supplementary figure inventory. Table S16. Full rectangular soil–depth matrix used for amplification and statistical analyses. Table S17. Shear-resistance sensitivity matrix at σ = 50 kPa. Table S18. Shear-resistance sensitivity matrix at σ = 100 kPa. Table S19. Shear-resistance sensitivity matrix at σ = 200 kPa. Table S20. Supplementary material quality-control checklist. Table S21. Crosswalk between main manuscript sections and supplementary support. Table S22. Recommended submission package for the manuscript and supplementary data.
