# Supplementary material for: Soil-Matrix-Dependent Root Reinforcement by Chrysopogon zizanioides: Direct Shear and Statistical Evidence from Andean Soils
Source: Plants (Basel). 2026 Jul 29;15(15):2328. doi: 10.3390/plants15152328 (PMC13468586; doi:10.3390/plants15152328)
Supplement: Supplementary file 1 [file plants-15-02328-s001.zip › plants-4435260-supplementary.pdf]

Supplementary Data

Soil-Matrix-Dependent Root Reinforcement by Chrysopogon zizanioides: Direct Shear and Statistical Evidence from Andean Soils

Camila Nickole Fernandez-Morocho, Jose Luis Chavez-Torres, Kunyong Zhang

This Supplementary Materials document provides the complete supporting data structure, derived calculations, statistical outputs, additional graphical diagnostics, and reproducibility notes for the manuscript. It is designed to allow independent verification of the root-content measurements, Mohr–Coulomb parameters, amplification calculations, one-way ANOVA screening, Tukey HSD comparisons, depth-dependent descriptive trends, and shear-resistance sensitivity analysis.

Contents of this Supplementary Data

This document provides the complete numerical datasets generated in this study, intended to support reproducibility and transparency of the reported results. The supplementary tables are organized as follows:

Section S1. Experimental design, variables, and workflow.

Section S2. Baseline soil matrices and Vetiver root-content distribution.

Section S3. Vetiver-rooted Mohr–Coulomb parameters.

Section S4. Root-induced amplification of c and φ.

Section S5. Statistical analysis and diagnostic checks.

Section S6. Tukey HSD post-hoc comparisons.

Section S7. Depth-dependent descriptive trends.

Section S8. Shear-resistance sensitivity analysis.

Section S9. Supplementary figure inventory and interpretation.

Section S10. Reproducibility notes and data availability.

The supplementary data are organized to mirror the sequence of the main manuscript. This prevents the supplementary file from becoming an unrelated appendix and ensures that each method used in the manuscript has a corresponding traceable dataset or verification output.

Section S1. Experimental design, variables, and workflow

The experimental design was structured to evaluate whether the mechanical contribution of Vetiver roots varies among soil matrices. The core comparison is between bare-soil parameters and Vetiver-rooted parameters across seven soil matrices and three depth intervals.

Figure S1. Experimental–statistical workflow.

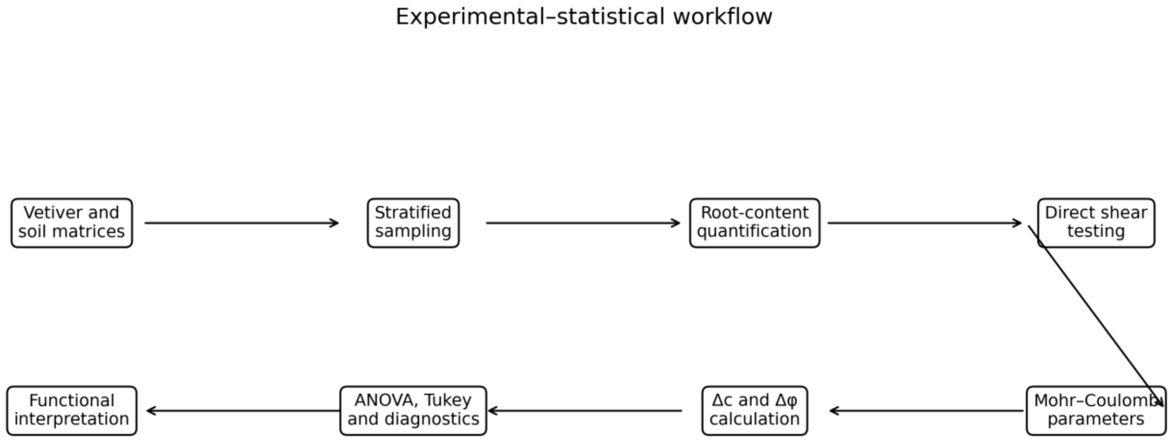

Workflow used to evaluate Vetiver-induced biomechanical reinforcement. The sequence links plant and soil selection, stratified sampling, root-content quantification, direct shear testing, Mohr–Coulomb parameter derivation, amplification analysis, statistical inference, and functional interpretation.

Table S1. Experimental variables and analytical role.

Variable / component	Notation	Values / levels	Purpose
Plant species	—	Chrysopogon zizanioides	Bioengineering species
Soil condition	—	Bare; Vetiver-rooted	Reference and root-affected comparison
Soil matrix	—	CL-ML, MH, CH, OH, OL, CL, ML	Matrix-dependent interpretation
Depth interval	d	0.00–0.50 m; 0.50–1.50 m; 1.50–2.00 m	Depth-based root–soil response
Root content	Rc	Dry root mass / dry soil mass × 100	Root presence descriptor
Normal stress	σ	50, 100, 200 kPa	Failure envelope construction
Shearing rate	—	1.2 mm/min	Controlled test condition
Primary mechanical parameters	c, φ	Apparent cohesion; internal friction angle	Mohr–Coulomb interpretation
Derived variables	Δc, Δφ, Δτ	Relative amplification and shear-resistance sensitivity	Response quantification
Statistical screening	ANOVA, Tukey HSD	One-way screening by soil type and depth	Statistical differentiation

S1.1. Data dictionary

Table S2. Minimum variable dictionary for reproducing the analysis.

Variable	Type	Definition	Unit / levels
soil_matrix	Categorical	USCS-based soil matrix identifier.	CL-ML, MH, CH, OH, OL, CL, ML
depth_interval	Categorical	Sampling depth interval for Vetiver-rooted specimens.	Upper, middle, deep
depth_midpoint	Numerical	Representative midpoint of each depth interval.	0.25, 1.00, 1.75 m
Rc	Numerical	Root-content percentage based on dry root mass and dry soil mass.	%
c_bare	Numerical	Apparent cohesion of the bare-soil reference condition.	kPa
phi_bare	Numerical	Internal friction angle of the bare-soil reference condition.	degrees
c_vetiver	Numerical	Apparent cohesion of the Vetiver-rooted specimen.	kPa
phi_vetiver	Numerical	Internal friction angle of the Vetiver-rooted specimen.	degrees
Delta_c	Numerical	Relative amplification of apparent cohesion.	%
Delta_phi	Numerical	Relative amplification of internal friction angle.	%
sigma	Numerical	Applied normal stress in the sensitivity calculation.	kPa
tau_bare	Numerical	Calculated shear resistance of the bare-soil condition.	kPa
tau_vetiver	Numerical	Calculated shear resistance of the Vetiver-rooted condition.	kPa
Delta_tau	Numerical	Relative change in calculated shear resistance.	%

Section S2. Baseline soil matrices and Vetiver root-content distribution

The bare-soil condition provided the reference state for the seven soil matrices. Root content was used as a descriptive indicator of root presence but was not interpreted as the sole predictor of reinforcement.

Table S3. Bare-soil Mohr–Coulomb parameters used as reference conditions.

Soil matrix	c bare (kPa)	φ bare (°)
CL-ML	25.77	24.34
MH	31.07	21.48
CH	50.70	14.57
OH	18.53	14.87
OL	2.69	29.49
CL	6.82	27.82
ML	16.96	26.10

Table S4. Vetiver root-content distribution by soil matrix and depth interval.

Soil matrix	Rc upper (%)	Rc middle (%)	Rc deep (%)	Mean Rc (%)	Pattern
CL-ML	0.95	0.23	0.14	0.44	Near-surface dominant
MH	0.95	0.23	0.21	0.46	Near-surface dominant
CH	0.92	0.19	0.19	0.43	Near-surface dominant
OH	0.31	0.22	0.21	0.25	Near-surface dominant
OL	0.23	0.14	0.11	0.16	Near-surface dominant
CL	0.23	0.14	0.10	0.16	Near-surface dominant
ML	0.23	0.11	0.10	0.15	Near-surface dominant

Note: Rc values are reported as percentages. Root content generally decreases with depth.

Figure S2. Vetiver root-content heatmap.

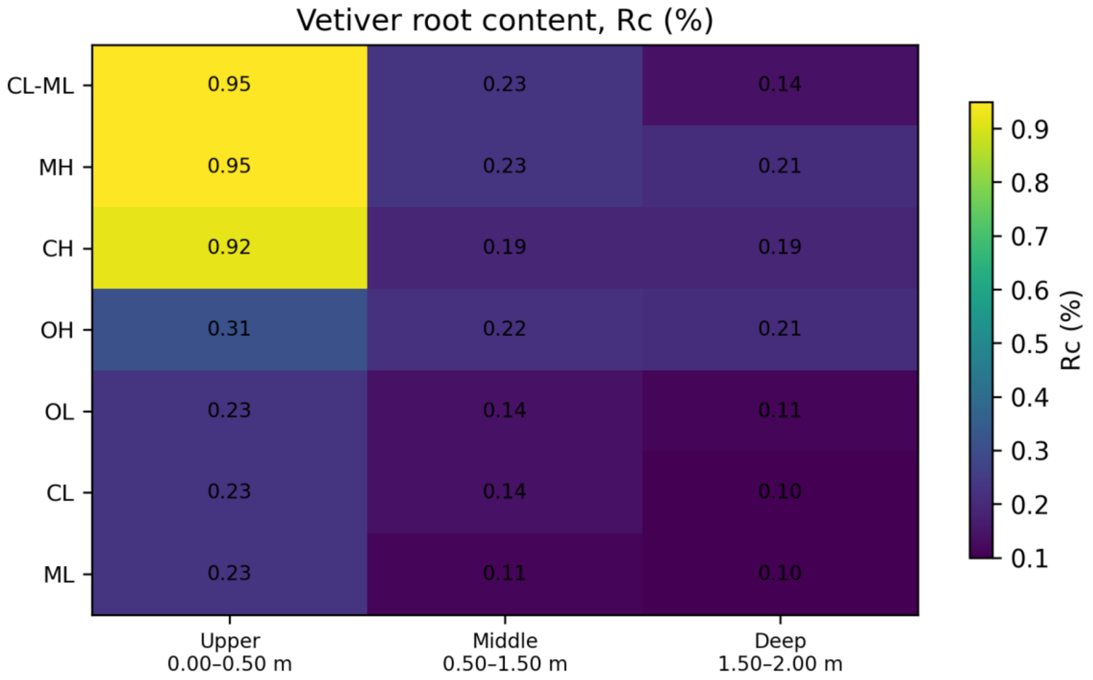

Root-content distribution across seven soil matrices and three depth intervals. The near-surface concentration is evident, but the highest Rc values do not automatically correspond to the highest mechanical amplification.

Section S3. Vetiver-rooted Mohr–Coulomb parameters

The Vetiver-rooted Mohr–Coulomb parameters are reported in full to allow independent verification of the amplification calculations and the shear-resistance sensitivity analysis.

Table S5. Vetiver-rooted Mohr–Coulomb parameters by soil matrix and depth interval.

Soil matrix	Depth interval	c Vetiver (kPa)	φ Vetiver (°)
CL-ML	0.00–0.50 m	26.78	27.41
CL-ML	0.50–1.50 m	27.78	28.31
CL-ML	1.50–2.00 m	29.55	28.47
MH	0.00–0.50 m	34.33	22.33
MH	0.50–1.50 m	34.83	23.65
MH	1.50–2.00 m	36.34	24.00
CH	0.00–0.50 m	51.70	16.43
CH	0.50–1.50 m	52.96	17.33
CH	1.50–2.00 m	56.01	17.50
OH	0.00–0.50 m	19.23	15.40
OH	0.50–1.50 m	19.33	15.91
OH	1.50–2.00 m	19.98	16.66
OL	0.00–0.50 m	4.13	30.01
OL	0.50–1.50 m	5.12	30.13
OL	1.50–2.00 m	5.38	30.22
CL	0.00–0.50 m	8.15	28.88
CL	0.50–1.50 m	8.66	28.94
CL	1.50–2.00 m	8.91	29.67
ML	0.00–0.50 m	20.49	27.31
ML	0.50–1.50 m	21.00	27.50
ML	1.50–2.00 m	22.25	27.85

Figure S3. Vetiver-rooted Mohr–Coulomb parameter heatmaps.

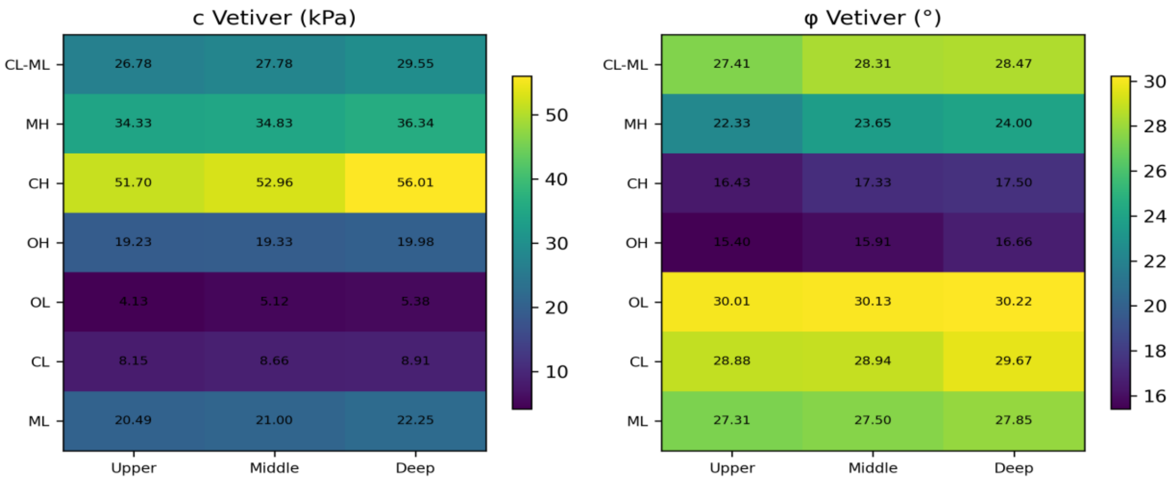

Heatmaps of depth-dependent apparent cohesion and internal friction angle for Vetiver-rooted specimens.

Figure S9. Mohr–Coulomb failure envelopes for bare and Vetiver-rooted conditions.

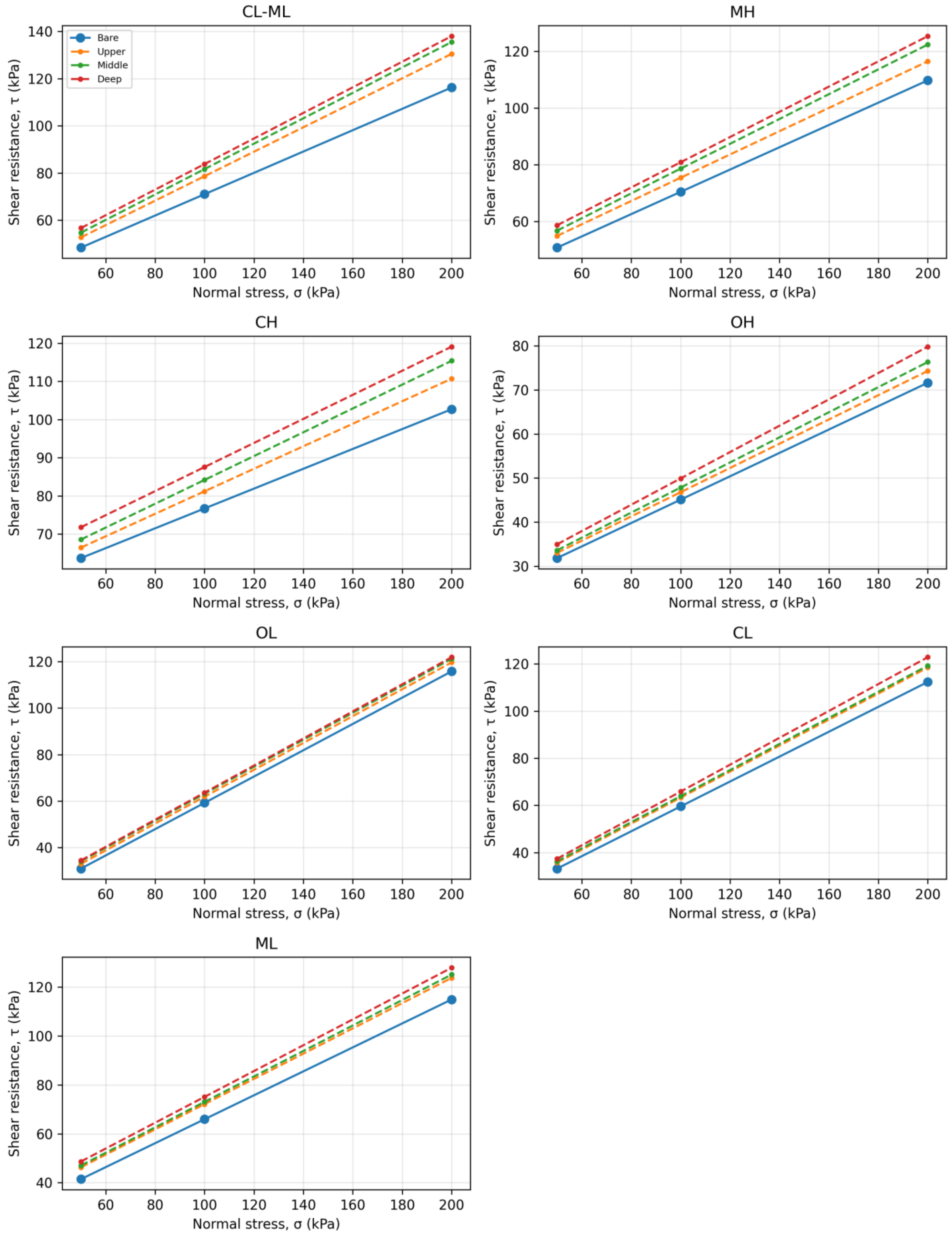

Calculated shear-resistance envelopes for the seven soil matrices under σ = 50, 100, and 200 kPa. The envelopes visually support the interpretation that Vetiver-induced reinforcement varies by soil matrix.

Section S4. Root-induced amplification of c and φ

The relative amplification values were calculated using the bare-soil condition as the denominator for each soil matrix. This section provides the complete Δc and Δφ matrix and the summary used in the main manuscript.

Table S6. Complete Vetiver-induced amplification matrix for apparent cohesion and internal friction angle.

Soil	Depth	c bare	c Vetiver	Δc (%)	φ bare	φ Vetiver	Δφ (%)
CL-ML	0.00–0.50 m	25.77	26.78	3.9	24.34	27.41	12.6
CL-ML	0.50–1.50 m	25.77	27.78	7.8	24.34	28.31	16.3
CL-ML	1.50–2.00 m	25.77	29.55	14.7	24.34	28.47	17.0
MH	0.00–0.50 m	31.07	34.33	10.5	21.48	22.33	4.0
MH	0.50–1.50 m	31.07	34.83	12.1	21.48	23.65	10.1
MH	1.50–2.00 m	31.07	36.34	17.0	21.48	24.00	11.7
CH	0.00–0.50 m	50.70	51.70	2.0	14.57	16.43	12.8
CH	0.50–1.50 m	50.70	52.96	4.5	14.57	17.33	18.9
CH	1.50–2.00 m	50.70	56.01	10.5	14.57	17.50	20.1
OH	0.00–0.50 m	18.53	19.23	3.8	14.87	15.40	3.6
OH	0.50–1.50 m	18.53	19.33	4.3	14.87	15.91	7.0
OH	1.50–2.00 m	18.53	19.98	7.8	14.87	16.66	12.0
OL	0.00–0.50 m	2.69	4.13	53.5	29.49	30.01	1.8
OL	0.50–1.50 m	2.69	5.12	90.3	29.49	30.13	2.2
OL	1.50–2.00 m	2.69	5.38	100.0	29.49	30.22	2.5
CL	0.00–0.50 m	6.82	8.15	19.5	27.82	28.88	3.8
CL	0.50–1.50 m	6.82	8.66	27.0	27.82	28.94	4.0
CL	1.50–2.00 m	6.82	8.91	30.6	27.82	29.67	6.6
ML	0.00–0.50 m	16.96	20.49	20.8	26.10	27.31	4.6
ML	0.50–1.50 m	16.96	21.00	23.8	26.10	27.50	5.4
ML	1.50–2.00 m	16.96	22.25	31.2	26.10	27.85	6.7

Note: Percentages are rounded to one decimal place.

Table S7. Summary of Vetiver-induced amplification by soil matrix.

Soil	Mean Δc (%)	Max Δc (%)	Mean Δφ (%)	Max Δφ (%)	Main pattern
CL-ML	8.8	14.7	15.3	17.0	High friction-angle response
MH	13.2	17.0	8.6	11.7	Balanced cohesion–friction response
CH	5.6	10.5	17.3	20.1	Highest friction-angle amplification
OH	5.3	7.8	7.5	12.0	Moderate response
OL	81.3	100.0	2.1	2.5	Highest relative cohesion amplification

Soil	Mean Δc (%)	Max Δc (%)	Mean Δφ (%)	Max Δφ (%)	Main pattern
CL	25.7	30.6	4.8	6.6	Moderate-to-high cohesion response
ML	25.3	31.2	5.6	6.7	Moderate-to-high cohesion response

Figure S4. Amplification heatmaps for Δc and Δφ.

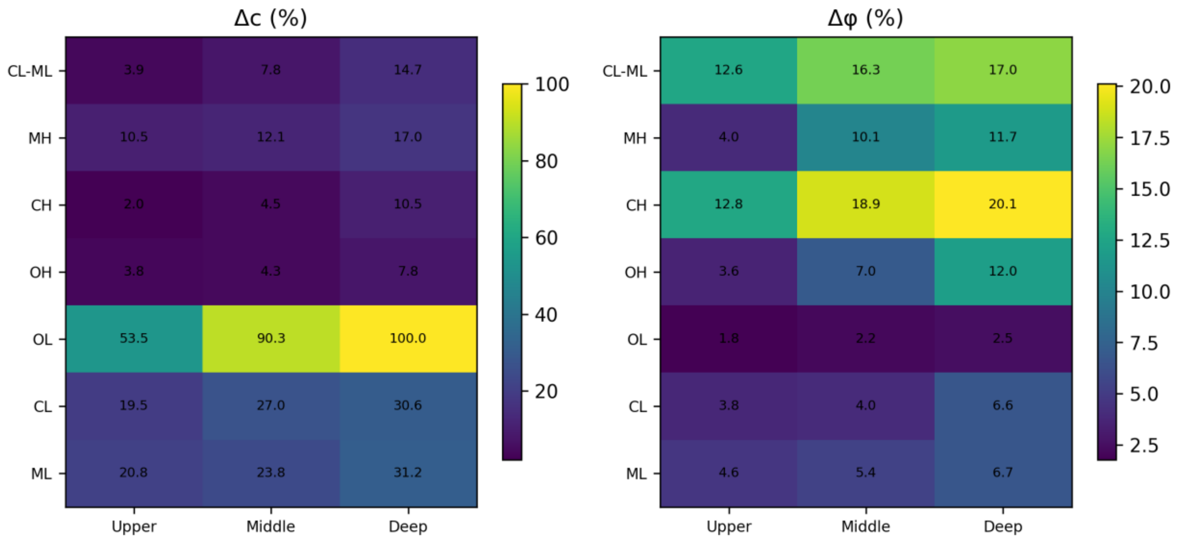

Relative changes in apparent cohesion and internal friction angle induced by Vetiver roots. OL shows the dominant relative cohesion response, whereas CH and CL-ML show stronger friction-angle amplification.

Figure S10. Depth profiles of Δc and Δφ.

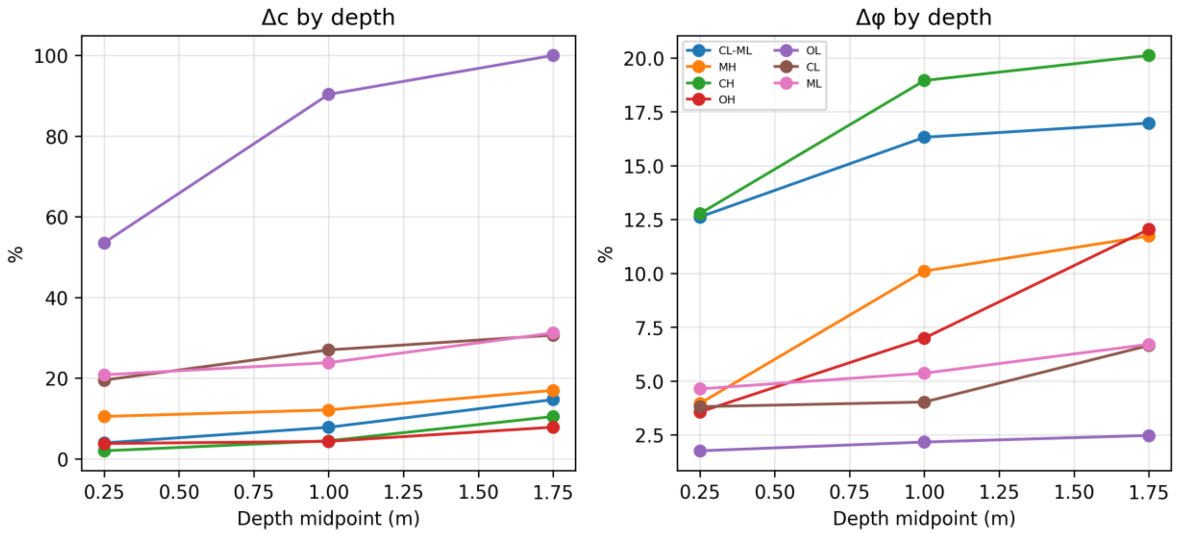

Depth-dependent amplification profiles for all soil matrices. The figure illustrates that soil-matrix effects are stronger than depth-only effects.

Section S5. Statistical analysis and diagnostic checks

Because each soil–depth cell had one amplification value, the statistical interpretation was limited to one-way ANOVA screening by soil type and by depth interval. The interaction term was not interpreted.

Table S8. One-way ANOVA screening results.

Response	Factor screened	F-value	p-value	η^2	Interpretation
Δc	Soil type	20.80	3.18e-06	0.899	Dominant
Δc	Depth interval	0.45	0.644	0.048	Not significant
Δφ	Soil type	10.90	0.000136	0.824	Dominant
Δφ	Depth interval	1.24	0.314	0.121	Not significant

Figure S11. Effect sizes for ANOVA screening models.

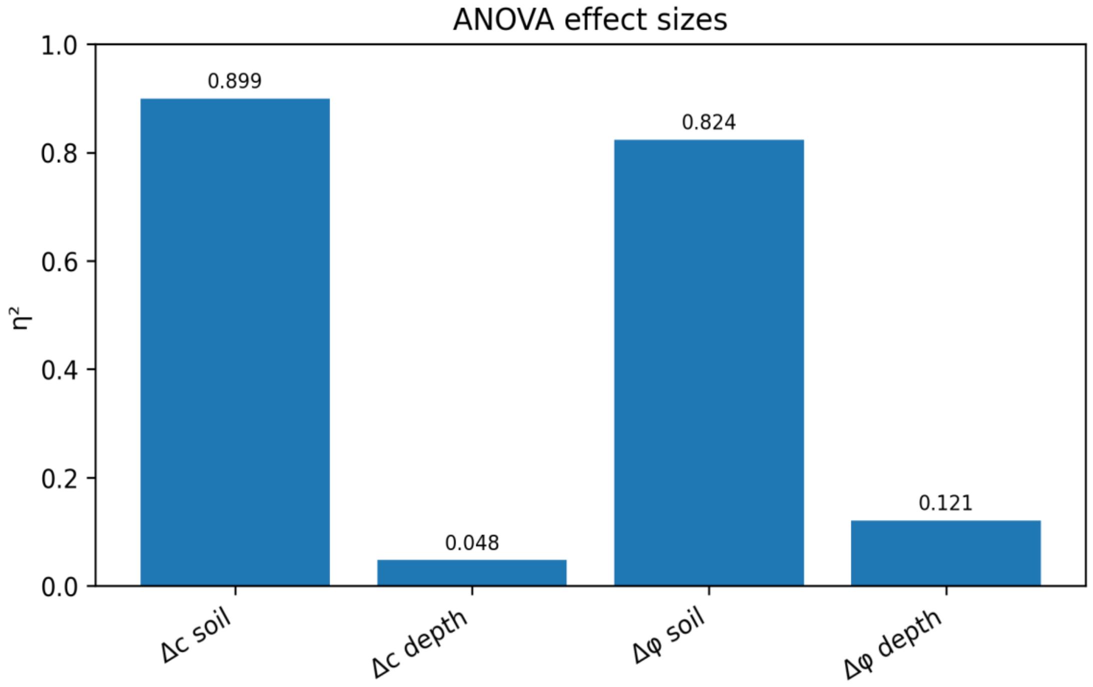

Effect-size comparison showing that soil type explains most of the variability in both Δc and Δφ, whereas depth interval alone explains a smaller proportion of variability.

Table S9. Diagnostic tests for the one-way ANOVA screening models.

Response	Model screened	Shapiro–Wilk p	Brown–Forsythe p	Diagnostic interpretation
Δc	Soil type	0.0034	0.375	Residual normality not satisfied; variance homogeneity not rejected
Δc	Depth interval	5.53e-05	0.864	Residual normality not satisfied; variance homogeneity not rejected
Δφ	Soil type	0.442	0.689	Diagnostics acceptable
Δφ	Depth interval	0.0839	0.622	Diagnostics acceptable

Figure S8. Diagnostic plots for soil-type ANOVA models.

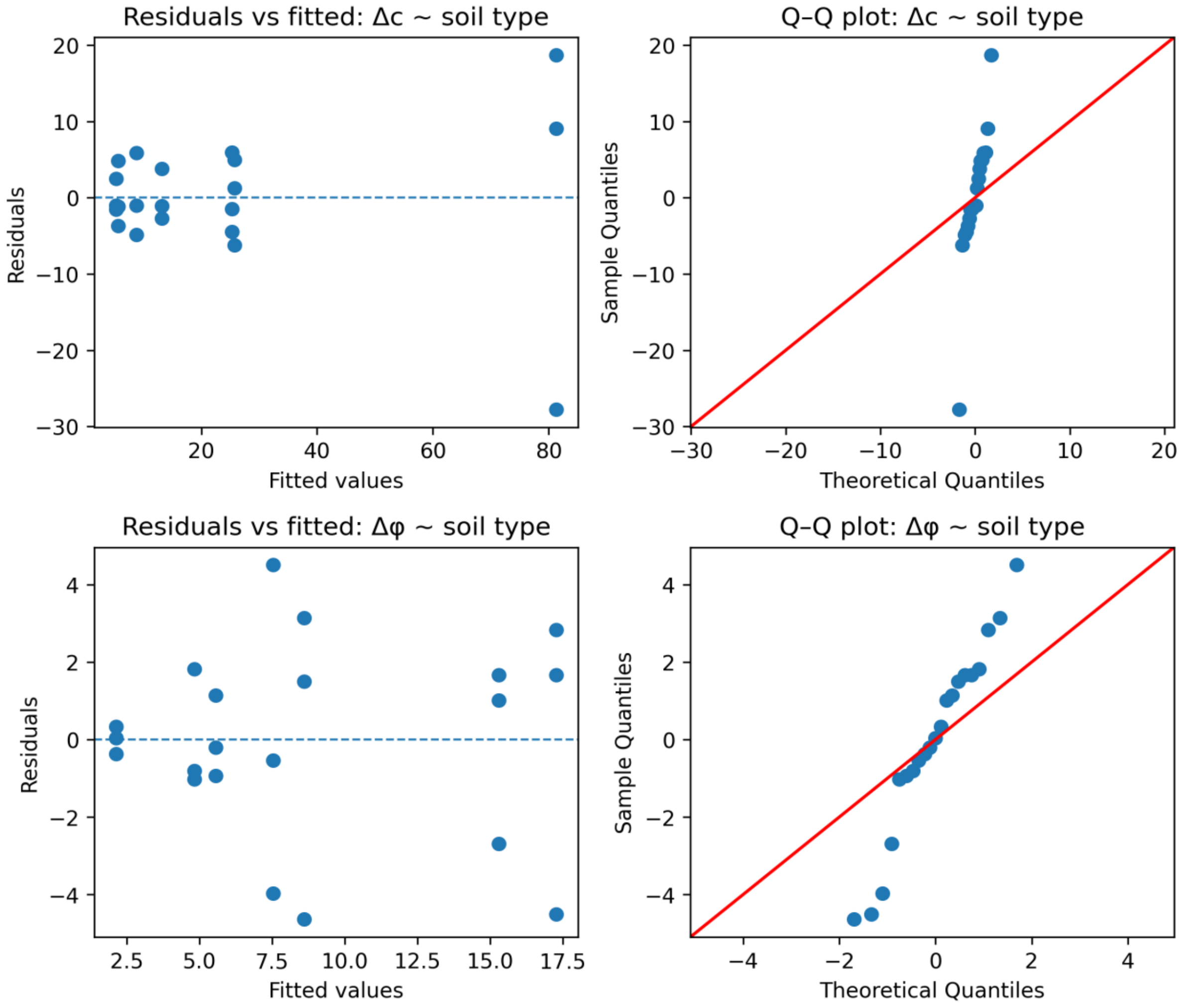

Residuals versus fitted values and Q–Q plots for the soil-type models. The Δc model is influenced by the extreme OL response, whereas Δφ shows more regular residual behaviour.

Section S6. Tukey HSD post-hoc comparisons

Tukey HSD comparisons were used to identify which soil matrices differed after the one-way ANOVA screening. Complete pairwise outputs are reported to support reproducibility.

Table S10. Complete Tukey HSD pairwise comparisons for Δc.

Contrast	Mean diff. (%)	95% CI (%)	Adjusted p-value	Result
CL – CH	20.07	-8.34 to 48.48	0.2624	ns
CL-ML – CH	3.16	-25.25 to 31.57	0.9997	ns
MH – CH	7.55	-20.86 to 35.96	0.9652	ns
ML – CH	19.64	-8.77 to 48.05	0.2830	ns
OH – CH	-0.33	-28.74 to 28.08	1.0000	ns
OL – CH	75.65	47.24 to 104.06	<0.0001	Significant
CL-ML – CL	-16.91	-45.32 to 11.50	0.4377	ns

Contrast	Mean diff. (%)	95% CI (%)	Adjusted p-value	Result
MH – CL	-12.52	-40.93 to 15.89	0.7379	ns
ML – CL	-0.43	-28.84 to 27.98	1.0000	ns
OH – CL	-20.40	-48.81 to 8.01	0.2476	ns
OL – CL	55.58	27.17 to 83.99	0.0002	Significant
MH – CL-ML	4.39	-24.02 to 32.80	0.9979	ns
ML – CL-ML	16.48	-11.93 to 44.89	0.4658	ns
OH – CL-ML	-3.49	-31.90 to 24.92	0.9994	ns
OL – CL-ML	72.49	44.08 to 100.90	<0.0001	Significant
ML – MH	12.09	-16.32 to 40.50	0.7660	ns
OH – MH	-7.88	-36.29 to 20.53	0.9576	ns
OL – MH	68.10	39.69 to 96.51	<0.0001	Significant
OH – ML	-19.97	-48.38 to 8.44	0.2673	ns
OL – ML	56.01	27.60 to 84.42	0.0002	Significant
OL – OH	75.98	47.57 to 104.39	<0.0001	Significant

Note: Mean differences are expressed as group2 minus group1. Significant OL contrasts support the interpretation of OL as a differentiated cohesion-amplification matrix.

Table S11. Complete Tukey HSD pairwise comparisons for $\Delta\varphi$.

Contrast	Mean diff. (%)	95% CI (%)	Adjusted p-value	Result
CL – CH	-12.44	-20.59 to -4.30	0.0019	Significant
CL-ML – CH	-1.98	-10.12 to 6.17	0.9776	ns
MH – CH	-8.68	-16.82 to -0.53	0.0336	Significant
ML – CH	-11.70	-19.85 to -3.56	0.0034	Significant
OH – CH	-9.74	-17.89 to -1.59	0.0149	Significant
OL – CH	-15.14	-23.28 to -6.99	0.0003	Significant
CL-ML – CL	10.47	2.32 to 18.62	0.0086	Significant
MH – CL	3.77	-4.38 to 11.92	0.6966	ns
ML – CL	0.74	-7.41 to 8.89	0.9999	ns
OH – CL	2.70	-5.45 to 10.85	0.9073	ns
OL – CL	-2.69	-10.84 to 5.46	0.9088	ns
MH – CL-ML	-6.70	-14.85 to 1.45	0.1419	ns
ML – CL-ML	-9.73	-17.88 to -1.58	0.0151	Significant
OH – CL-ML	-7.77	-15.91 to 0.38	0.0664	ns
OL – CL-ML	-13.16	-21.31 to -5.01	0.0011	Significant
ML – MH	-3.03	-11.18 to 5.12	0.8547	ns
OH – MH	-1.07	-9.21 to 7.08	0.9992	ns
OL – MH	-6.46	-14.61 to 1.69	0.1669	ns
OH – ML	1.96	-6.18 to 10.11	0.9783	ns
OL – ML	-3.43	-11.58 to 4.72	0.7738	ns
OL – OH	-5.40	-13.54 to 2.75	0.3254	ns

Note: The significant contrasts identify CH and CL-ML as dominant friction-angle response matrices.

Figure S12. Forest plots of significant Tukey HSD contrasts.

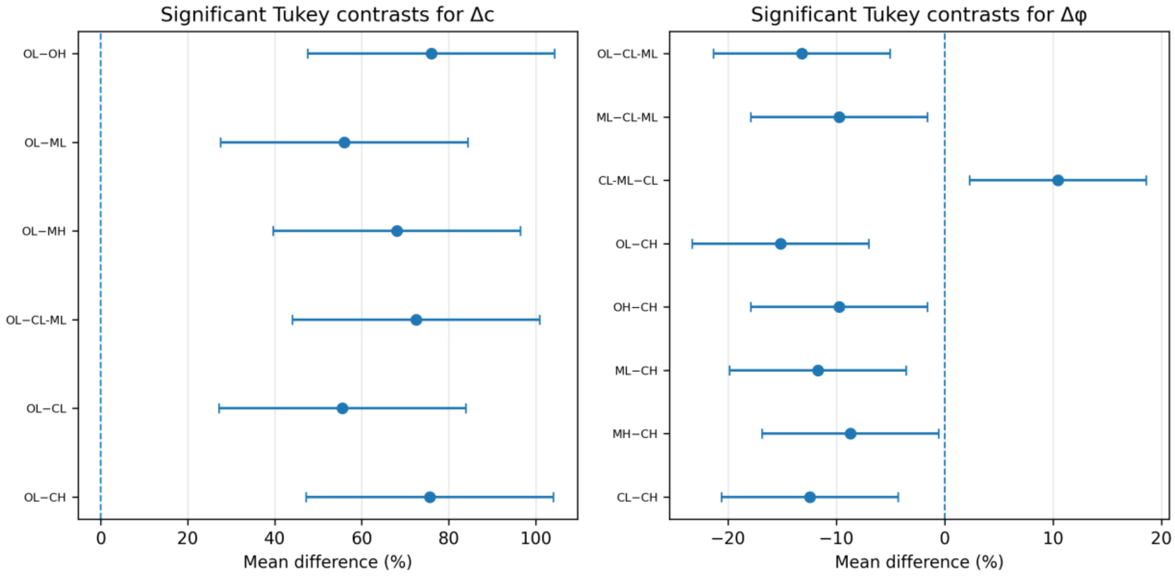

Significant Tukey contrasts for Δc and Δφ. The Δc contrasts are dominated by OL, whereas the Δφ contrasts highlight CH and CL-ML.

Figure S5. Soil-matrix group means and 95% confidence intervals.

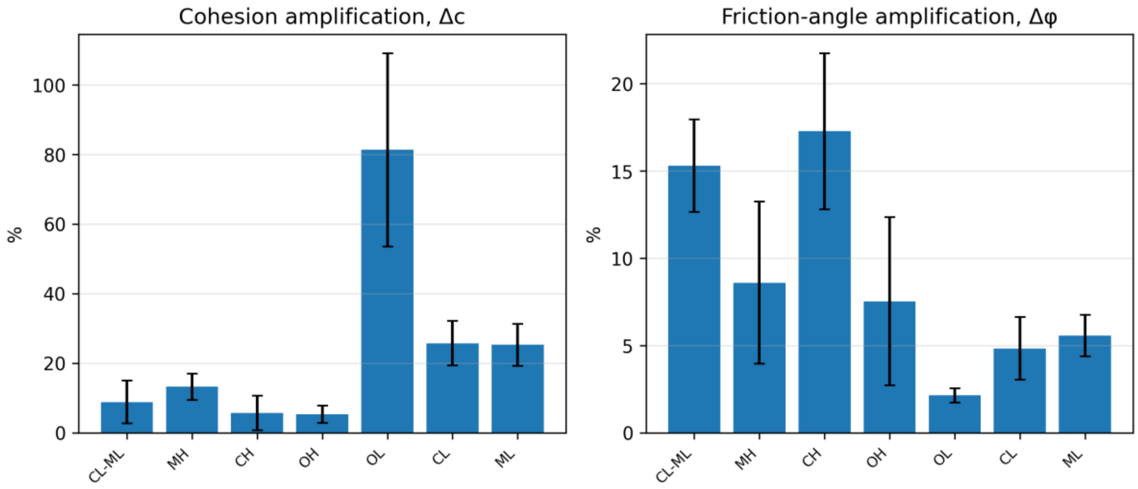

Group means and approximate 95% confidence intervals for Δc and Δφ. These plots visually support the post-hoc interpretation.

Section S7. Depth-dependent descriptive trends

The focal matrices OL, MH, and CH were evaluated through simple descriptive depth trends. These regressions are not used as predictive models because only three depth points are available per soil matrix.

Table S12. Descriptive depth-dependent trends for focal soil matrices.

Soil	Parameter	Linear trend	R^2	p-value	Use
OL	c	c(z) = 4.043 + 0.833z	0.898	0.207	Descriptive only
OL	φ	φ(z) = 29.980 + 0.140z	0.993	0.052	Descriptive only
OL	Δc	Δc(z) = 50.310 + 30.979z	0.898	0.207	Descriptive only

Soil	Parameter	Linear trend	R²	p-value	Use
OL	Δφ	Δφ(z) = 1.662 + 0.475z	0.993	0.052	Descriptive only
MH	c	c(z) = 33.827 + 1.340z	0.922	0.180	Descriptive only
MH	φ	φ(z) = 22.213 + 1.113z	0.899	0.206	Descriptive only
MH	Δc	Δc(z) = 8.872 + 4.313z	0.922	0.180	Descriptive only
MH	Δφ	Δφ(z) = 3.414 + 5.183z	0.899	0.206	Descriptive only
CH	c	c(z) = 50.683 + 2.873z	0.946	0.150	Descriptive only
CH	φ	φ(z) = 16.373 + 0.713z	0.866	0.239	Descriptive only
CH	Δc	Δc(z) = -0.033 + 5.667z	0.946	0.150	Descriptive only
CH	Δφ	Δφ(z) = 12.377 + 4.896z	0.866	0.239	Descriptive only

Note: z is the representative depth midpoint in metres.

Figure S6. Depth-dependent trends in focal soil matrices.

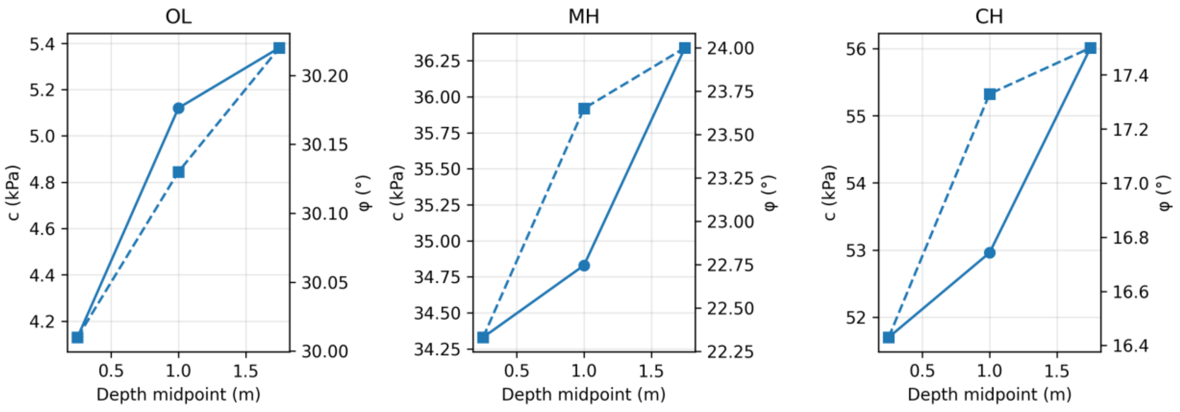

Depth-dependent c and φ trends for OL, MH, and CH.

Section S8. Shear-resistance sensitivity analysis

The sensitivity analysis recalculates shear resistance under the same normal stresses used in the direct shear tests. This allows the practical mechanical relevance of Δc and Δφ to be assessed through their combined influence on τ.

Table S13. Complete shear-resistance sensitivity matrix.

Soil	Depth	σ (kPa)	τ bare (kPa)	τ Vetiver (kPa)	Δτ (%)
CL-ML	0.00–0.50 m	50	48.39	52.71	8.9
CL-ML	0.00–0.50 m	100	71.01	78.64	10.7
CL-ML	0.00–0.50 m	200	116.24	130.49	12.3
CL-ML	0.50–1.50 m	50	48.39	54.71	13.1
CL-ML	0.50–1.50 m	100	71.01	81.65	15.0
CL-ML	0.50–1.50 m	200	116.24	135.51	16.6
CL-ML	1.50–2.00 m	50	48.39	56.66	17.1
CL-ML	1.50–2.00 m	100	71.01	83.78	18.0
CL-ML	1.50–2.00 m	200	116.24	138.01	18.7
MH	0.00–0.50 m	50	50.75	54.87	8.1
MH	0.00–0.50 m	100	70.42	75.40	7.1

Soil	Depth	σ (kPa)	τ bare (kPa)	τ Vetiver (kPa)	Δτ (%)
MH	0.00–0.50 m	200	109.77	116.48	6.1
MH	0.50–1.50 m	50	50.75	56.73	11.8
MH	0.50–1.50 m	100	70.42	78.62	11.6
MH	0.50–1.50 m	200	109.77	122.42	11.5
MH	1.50–2.00 m	50	50.75	58.60	15.5
MH	1.50–2.00 m	100	70.42	80.86	14.8
MH	1.50–2.00 m	200	109.77	125.39	14.2
CH	0.00–0.50 m	50	63.70	66.44	4.3
CH	0.00–0.50 m	100	76.69	81.19	5.9
CH	0.00–0.50 m	200	102.68	110.68	7.8
CH	0.50–1.50 m	50	63.70	68.56	7.6
CH	0.50–1.50 m	100	76.69	84.16	9.7
CH	0.50–1.50 m	200	102.68	115.37	12.4
CH	1.50–2.00 m	50	63.70	71.77	12.7
CH	1.50–2.00 m	100	76.69	87.54	14.1
CH	1.50–2.00 m	200	102.68	119.07	16.0
OH	0.00–0.50 m	50	31.81	33.00	3.8
OH	0.00–0.50 m	100	45.08	46.77	3.8
OH	0.00–0.50 m	200	71.63	74.32	3.7
OH	0.50–1.50 m	50	31.81	33.58	5.6
OH	0.50–1.50 m	100	45.08	47.83	6.1
OH	0.50–1.50 m	200	71.63	76.34	6.6
OH	1.50–2.00 m	50	31.81	34.94	9.9
OH	1.50–2.00 m	100	45.08	49.91	10.7
OH	1.50–2.00 m	200	71.63	79.83	11.4
OL	0.00–0.50 m	50	30.97	33.01	6.6
OL	0.00–0.50 m	100	59.24	61.89	4.5
OL	0.00–0.50 m	200	115.80	119.65	3.3
OL	0.50–1.50 m	50	30.97	34.14	10.2
OL	0.50–1.50 m	100	59.24	63.16	6.6
OL	0.50–1.50 m	200	115.80	121.20	4.7
OL	1.50–2.00 m	50	30.97	34.50	11.4
OL	1.50–2.00 m	100	59.24	63.63	7.4
OL	1.50–2.00 m	200	115.80	121.88	5.2
CL	0.00–0.50 m	50	33.20	35.73	7.6
CL	0.00–0.50 m	100	59.59	63.31	6.2
CL	0.00–0.50 m	200	112.36	118.46	5.4
CL	0.50–1.50 m	50	33.20	36.31	9.3
CL	0.50–1.50 m	100	59.59	63.95	7.3
CL	0.50–1.50 m	200	112.36	119.25	6.1
CL	1.50–2.00 m	50	33.20	37.39	12.6
CL	1.50–2.00 m	100	59.59	65.88	10.6
CL	1.50–2.00 m	200	112.36	122.85	9.3

Soil	Depth	σ (kPa)	τ bare (kPa)	τ Vetiver (kPa)	Δτ (%)
ML	0.00–0.50 m	50	41.45	46.31	11.7
ML	0.00–0.50 m	100	65.95	72.13	9.4
ML	0.00–0.50 m	200	114.94	123.76	7.7
ML	0.50–1.50 m	50	41.45	47.03	13.4
ML	0.50–1.50 m	100	65.95	73.06	10.8
ML	0.50–1.50 m	200	114.94	125.11	8.9
ML	1.50–2.00 m	50	41.45	48.67	17.4
ML	1.50–2.00 m	100	65.95	75.09	13.9
ML	1.50–2.00 m	200	114.94	127.92	11.3

Note: $\tau = c + \sigma \tan(\varphi)$, with φ expressed in degrees.

Table S14. Mean shear-resistance sensitivity by soil matrix and normal stress.

Soil	Mean Δτ 50 kPa (%)	Mean Δτ 100 kPa (%)	Mean Δτ 200 kPa (%)	Global mean Δτ (%)	Sensitivity interpretation
CL-ML	13.0	14.6	15.9	14.5	Highest overall shear-resistance sensitivity
MH	11.8	11.2	10.6	11.2	Balanced improvement
CH	8.2	9.9	12.0	10.1	Friction-angle-driven improvement
OH	6.4	6.9	7.3	6.8	Moderate response
OL	9.4	6.2	4.4	6.7	High Δc but moderate Δτ response
CL	9.9	8.0	7.0	8.3	Moderate cohesion-driven response
ML	14.2	11.3	9.3	11.6	Higher sensitivity at low normal stress

Figure S7. Mean shear-resistance sensitivity heatmap.

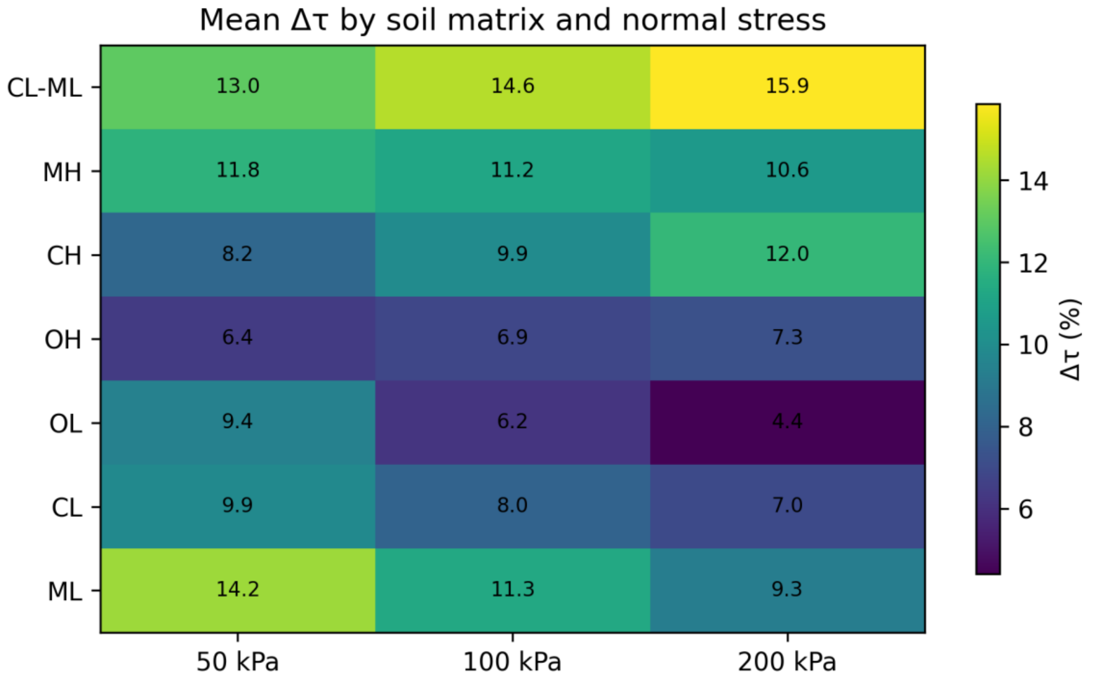

Mean Δτ values under normal stresses of 50, 100, and 200 kPa.

Figure S13. Stress-dependent shear-resistance sensitivity profiles.

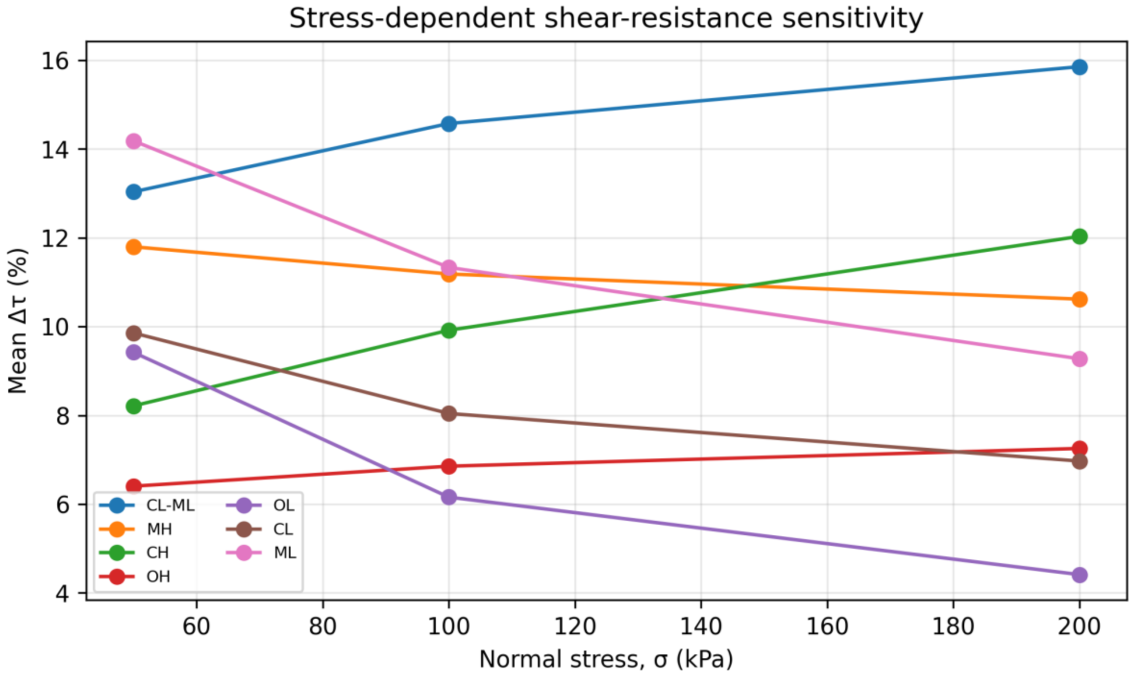

Mean Δτ profiles showing how the influence of Vetiver-modified parameters changes with normal stress.

Section S9. Supplementary figure inventory and interpretation

Table S15. Supplementary figure inventory.

Figure	Content	Role
Figure S1	Experimental–statistical workflow	Documents the study sequence.
Figure S2	Root-content heatmap	Supports interpretation of Rc distribution.
Figure S3	Mohr–Coulomb parameter heatmaps	Documents c and φ matrices for Vetiver-rooted specimens.
Figure S4	Amplification heatmaps	Documents Δc and Δφ matrices.
Figure S5	Group means and confidence intervals	Supports statistical group interpretation.
Figure S6	Depth-dependent trends	Supports descriptive trend interpretation for focal matrices.
Figure S7	Sensitivity heatmap	Documents mean Δτ values by soil and stress.
Figure S8	Diagnostic plots	Supports ANOVA assumption discussion.
Figure S9	Failure envelopes	Visualizes bare and Vetiver-rooted τ–σ responses.
Figure S10	Amplification depth profiles	Shows soil-specific trends across depth intervals.
Figure S11	ANOVA effect sizes	Visualizes η^2 values.
Figure S12	Tukey forest plots	Visualizes significant post-hoc contrasts.
Figure S13	Sensitivity profiles	Visualizes stress-dependent Δτ behaviour.

Together, the supplementary figures provide graphical support for the central interpretation of the manuscript: Vetiver-induced reinforcement is not a uniform plant effect, but a soil-matrix-dependent response. OL is mechanically distinctive in relative cohesion amplification, CH is distinctive in friction-angle amplification, and MH represents an intermediate balanced response.

Section S10. Reproducibility notes and data availability

The structured data table should be stored in rectangular format to reproduce all results. The minimum required variables are soil matrix, depth interval, depth midpoint, root content, bare-soil cohesion, bare-soil friction angle, Vetiver-rooted cohesion, Vetiver-rooted friction angle, Δc, $\Delta\varphi$, normal stress, τ bare, τ Vetiver, and $\Delta\tau$.

The statistical outputs in this supplementary material were calculated from the 21-row soil–depth amplification matrix. The one-way screening approach was selected because replication within each soil–depth cell was not available. Therefore, interaction effects were not interpreted.

The original data supporting the findings of the study are available in the Zenodo repository reported in the Data Availability Statement of the main manuscript. The supplementary tables and figures provide a transparent record of the calculations used in the manuscript.

Supplementary References

S1. Ihaka, R.; Gentleman, R. R: A Language for Data Analysis and Graphics. Journal of Computational and Graphical Statistics 1996, 5, 299–314. https://doi.org/10.1080/10618600.1996.10474713.

S2. Shapiro, S.S.; Wilk, M.B. An Analysis of Variance Test for Normality (Complete Samples). Biometrika 1965, 52, 591–611. https://doi.org/10.1093/biomet/52.3-4.591.

S3. Brown, M.B.; Forsythe, A.B. Robust Tests for the Equality of Variances. Journal of the American Statistical Association 1974, 69, 364–367. https://doi.org/10.1080/01621459.1974.10482955.

Appendix S1. Full rectangular dataset for independent verification

This appendix provides the complete 21-row rectangular matrix used to calculate the amplification and statistical analyses. It combines the soil matrix, depth interval, root content, bare-soil parameters, Vetiver-rooted parameters, and derived response variables in a single table.

Table S16. Full rectangular soil–depth matrix used for amplification and statistical analyses.

Soil	Depth	z (m)	Rc (%)	c bare	φ bare	c Vetiver	φ Vetiver	Δc (%)	Δφ (%)
CL-ML	0.00–0.50 m	0.25	0.95	25.77	24.34	26.78	27.41	3.9	12.6
CL-ML	0.50–1.50 m	1.00	0.23	25.77	24.34	27.78	28.31	7.8	16.3
CL-ML	1.50–2.00 m	1.75	0.14	25.77	24.34	29.55	28.47	14.7	17.0
MH	0.00–0.50 m	0.25	0.95	31.07	21.48	34.33	22.33	10.5	4.0
MH	0.50–1.50 m	1.00	0.23	31.07	21.48	34.83	23.65	12.1	10.1
MH	1.50–2.00 m	1.75	0.21	31.07	21.48	36.34	24.00	17.0	11.7
CH	0.00–0.50 m	0.25	0.92	50.70	14.57	51.70	16.43	2.0	12.8
CH	0.50–1.50 m	1.00	0.19	50.70	14.57	52.96	17.33	4.5	18.9
CH	1.50–2.00 m	1.75	0.19	50.70	14.57	56.01	17.50	10.5	20.1
OH	0.00–0.50 m	0.25	0.31	18.53	14.87	19.23	15.40	3.8	3.6
OH	0.50–1.50 m	1.00	0.22	18.53	14.87	19.33	15.91	4.3	7.0
OH	1.50–2.00 m	1.75	0.21	18.53	14.87	19.98	16.66	7.8	12.0
OL	0.00–0.50 m	0.25	0.23	2.69	29.49	4.13	30.01	53.5	1.8
OL	0.50–1.50 m	1.00	0.14	2.69	29.49	5.12	30.13	90.3	2.2
OL	1.50–2.00 m	1.75	0.11	2.69	29.49	5.38	30.22	100.0	2.5
CL	0.00–0.50 m	0.25	0.23	6.82	27.82	8.15	28.88	19.5	3.8
CL	0.50–1.50 m	1.00	0.14	6.82	27.82	8.66	28.94	27.0	4.0
CL	1.50–2.00 m	1.75	0.10	6.82	27.82	8.91	29.67	30.6	6.6
ML	0.00–0.50 m	0.25	0.23	16.96	26.10	20.49	27.31	20.8	4.6
ML	0.50–1.50 m	1.00	0.11	16.96	26.10	21.00	27.50	23.8	5.4
ML	1.50–2.00 m	1.75	0.10	16.96	26.10	22.25	27.85	31.2	6.7

Note: This table is the minimum dataset needed to reproduce Tables S6–S12.

Appendix S2. Condensed shear-resistance matrices

The following matrices provide condensed τ and Δτ values by soil matrix, depth interval, and normal stress. These tables are derived from the Mohr–Coulomb equation and support the sensitivity analysis reported in the main manuscript.

Table S17. Shear-resistance sensitivity matrix at σ = 50 kPa.

Soil	Depth	τ bare (kPa)	τ Vetiver (kPa)	Δτ (%)
CL-ML	0.00–0.50 m	48.39	52.71	8.9
CL-ML	0.50–1.50 m	48.39	54.71	13.1
CL-ML	1.50–2.00 m	48.39	56.66	17.1
MH	0.00–0.50 m	50.75	54.87	8.1
MH	0.50–1.50 m	50.75	56.73	11.8
MH	1.50–2.00 m	50.75	58.60	15.5
CH	0.00–0.50 m	63.70	66.44	4.3
CH	0.50–1.50 m	63.70	68.56	7.6
CH	1.50–2.00 m	63.70	71.77	12.7
OH	0.00–0.50 m	31.81	33.00	3.8
OH	0.50–1.50 m	31.81	33.58	5.6
OH	1.50–2.00 m	31.81	34.94	9.9
OL	0.00–0.50 m	30.97	33.01	6.6
OL	0.50–1.50 m	30.97	34.14	10.2
OL	1.50–2.00 m	30.97	34.50	11.4
CL	0.00–0.50 m	33.20	35.73	7.6
CL	0.50–1.50 m	33.20	36.31	9.3
CL	1.50–2.00 m	33.20	37.39	12.6
ML	0.00–0.50 m	41.45	46.31	11.7
ML	0.50–1.50 m	41.45	47.03	13.4
ML	1.50–2.00 m	41.45	48.67	17.4

Note: Values were calculated with σ = 50 kPa.

Table S18. Shear-resistance sensitivity matrix at σ = 100 kPa.

Soil	Depth	τ bare (kPa)	τ Vetiver (kPa)	Δτ (%)
CL-ML	0.00–0.50 m	71.01	78.64	10.7
CL-ML	0.50–1.50 m	71.01	81.65	15.0
CL-ML	1.50–2.00 m	71.01	83.78	18.0
MH	0.00–0.50 m	70.42	75.40	7.1
MH	0.50–1.50 m	70.42	78.62	11.6
MH	1.50–2.00 m	70.42	80.86	14.8
CH	0.00–0.50 m	76.69	81.19	5.9
CH	0.50–1.50 m	76.69	84.16	9.7
CH	1.50–2.00 m	76.69	87.54	14.1
OH	0.00–0.50 m	45.08	46.77	3.8
OH	0.50–1.50 m	45.08	47.83	6.1
OH	1.50–2.00 m	45.08	49.91	10.7
OL	0.00–0.50 m	59.24	61.89	4.5
OL	0.50–1.50 m	59.24	63.16	6.6
OL	1.50–2.00 m	59.24	63.63	7.4
CL	0.00–0.50 m	59.59	63.31	6.2
CL	0.50–1.50 m	59.59	63.95	7.3
CL	1.50–2.00 m	59.59	65.88	10.6
ML	0.00–0.50 m	65.95	72.13	9.4
ML	0.50–1.50 m	65.95	73.06	10.8
ML	1.50–2.00 m	65.95	75.09	13.9

Note: Values were calculated with σ = 100 kPa.

Table S19. Shear-resistance sensitivity matrix at σ = 200 kPa.

Soil	Depth	τ bare (kPa)	τ Vetiver (kPa)	Δτ (%)
CL-ML	0.00–0.50 m	116.24	130.49	12.3
CL-ML	0.50–1.50 m	116.24	135.51	16.6
CL-ML	1.50–2.00 m	116.24	138.01	18.7
MH	0.00–0.50 m	109.77	116.48	6.1
MH	0.50–1.50 m	109.77	122.42	11.5
MH	1.50–2.00 m	109.77	125.39	14.2
CH	0.00–0.50 m	102.68	110.68	7.8
CH	0.50–1.50 m	102.68	115.37	12.4
CH	1.50–2.00 m	102.68	119.07	16.0
OH	0.00–0.50 m	71.63	74.32	3.7
OH	0.50–1.50 m	71.63	76.34	6.6
OH	1.50–2.00 m	71.63	79.83	11.4
OL	0.00–0.50 m	115.80	119.65	3.3
OL	0.50–1.50 m	115.80	121.20	4.7
OL	1.50–2.00 m	115.80	121.88	5.2
CL	0.00–0.50 m	112.36	118.46	5.4
CL	0.50–1.50 m	112.36	119.25	6.1
CL	1.50–2.00 m	112.36	122.85	9.3
ML	0.00–0.50 m	114.94	123.76	7.7
ML	0.50–1.50 m	114.94	125.11	8.9
ML	1.50–2.00 m	114.94	127.92	11.3

Note: Values were calculated with σ = 200 kPa.

Appendix S3. Pre-submission quality-control checklist

Table S20. Supplementary material quality-control checklist.

Quality-control item	Status
Main manuscript and Supplementary title are consistent	Checked
All supplementary tables use Vetiver-only data	Checked
No Salix, FracRoot, Double-Wedge, or canal-slope data retained	Checked
Seven soil matrices are retained: CL-ML, MH, CH, OH, OL, CL, ML	Checked
Three depth intervals are retained: upper, middle, deep	Checked
Bare-soil values correspond to the reference state used in the main manuscript	Checked
Vetiver-rooted c and φ values correspond to the main manuscript	Checked
Δc and Δφ are calculated from the bare and Vetiver-rooted values	Checked
ANOVA is described as one-way screening, not a factorial interaction model	Checked
Tukey HSD outputs are reported completely for Δc and Δφ	Checked
Sensitivity results are calculated from $\tau = c + \sigma \tan(\varphi)$	Checked
Figures support rather than duplicate the main manuscript narrative	Checked
Data availability is linked to the repository specified in the main manuscript	Checked

Appendix S4. Crosswalk between the main manuscript and the Supplementary Materials

Table S21 links the main manuscript sections with the supplementary tables and figures. This crosswalk is included to facilitate editorial review and to help readers verify where each calculation or graphical output is documented.

Table S21. Crosswalk between main manuscript sections and supplementary support.

Main manuscript location	Content verified	Supplementary support
Materials and Methods 2.1	Experimental workflow	Figure S1; Table S1
Materials and Methods 2.2	Bare-soil matrices	Table S3
Materials and Methods 2.5	Root-content quantification	Table S4; Figure S2
Materials and Methods 2.6–2.7	Direct shear and Mohr–Coulomb parameters	Table S5; Figure S3; Figure S9
Materials and Methods 2.8	Δc and $\Delta \varphi$ calculations	Table S6; Table S7; Figure S4; Figure S10
Materials and Methods 2.9	ANOVA, diagnostics, Tukey HSD	Tables S8–S11; Figures S5, S8, S11, S12
Materials and Methods 2.10	Depth-dependent descriptive trends	Table S12; Figure S6
Materials and Methods 2.11	Shear-resistance sensitivity	Tables S13–S19; Figures S7 and S13
Results 3.8	Integrated functional interpretation	Table S15; Table S20

Appendix S5. Recommended submission package

For final submission, the main manuscript, supplementary file, dataset, and figure files should be kept consistent. Table S22 summarizes the recommended submission package and the purpose of each file.

Table S22. Recommended submission package for the manuscript and supplementary data.

File	Purpose	Submission note
Main manuscript (.docx)	Article text, main tables, main figures, declarations	Must cite Supplementary Materials where appropriate
Supplementary Materials (.docx)	Supporting datasets, statistical outputs, additional figures	This file
Dataset (.csv or .xlsx)	Machine-readable soil–depth matrix and sensitivity table	Should match Tables S16–S19
Figures (.tif/.png)	High-resolution figures for production	Minimum 300 dpi
R or Python script (.R/.py)	Reproducible calculation of Δc, $\Delta \varphi$, ANOVA, Tukey, and $\Delta \tau$	Recommended for Zenodo
Readme file (.txt)	Brief explanation of variables and calculation sequence	Should match Table S2
